# Factors affecting the viability of probiotics in various food products: a review of current knowledge

**DOI:** 10.3389/fmicb.2026.1893153

**Published:** 2026-07-14

**Authors:** Ilkin Sengun, Aysegul Kirmizigul Peker, Heena Sharma, Nikheel Bhojraj Rathod, Tanaji G. Kudre, Andrzej Szymkowiak, Piotr Kulawik, Giulia Tabanelli, Duygu Ağagündüz, Ferenc Budán, Fatih Ozogul

**Affiliations:** 1 Food Engineering Department, Engineering Faculty, Ege University, Izmir, Bornova, Türkiye; 2 Food Technology Lab, Dairy Technology Division, ICAR-National Dairy Research Institute, Karnal, Haryana, India; 3 Department of Meat and Marine Sciences, CSIR-Central Food Technological Research Institute, Mysuru, India; 4 Department of Commerce and Marketing, Institute of Marketing, Poznań University of Economics and Business, Poznań, Poland; 5 Department of Animal Products Technology, Faculty of Food Technology, University of Agriculture, Kraków, Poland; 6 Department of Agricultural and Food Sciences, University of Bologna, Bologna, Italy; 7 Department of Nutrition and Dietetics, Gazi University, Ankara, Türkiye; 8 Medical School, Institute of Physiology, University of Pécs, Pécs, Hungary; 9 Department of Seafood Processing Technology, Faculty of Fisheries, Cukurova University, Adana, Türkiye

**Keywords:** fermented foods, food functionality, microencapsulation, non-thermal technologies, probiotic viability

## Abstract

This review comparatively evaluates the quantitative effects of non-thermal processing technologies on probiotic survival, metabolic stability, and functional performance across different strains and food matrices. A literature search identified relevant peer-reviewed studies, from which eligible original research articles were included for detailed synthesis. Recent quantitative evidence indicates that non-thermal technologies can enhance probiotic viability and functionality under controlled conditions. For example, ultrasound-assisted fermentation in a millet-based probiotic beverage containing *Lactiplantibacillus plantarum* reportedly reduced fermentation time by up to 2.6-fold and improved probiotic viability by approximately 1–1.5 log CFU/mL under specific treatment conditions. Similarly, high hydrostatic pressure treatments at 200–300 MPa maintained probiotic counts in selected food systems, while pulsed electric field-assisted pretreatment within a specific spray-drying protocol elevated intracellular trehalose levels to approximately 100 mM and achieved nearly 75% survival. Microencapsulation strategies and emerging biological approaches, including bacteriophage-related adaptations, further contribute to stress protection during processing. Overall, non-thermal technologies represent promising alternatives to conventional treatments; however, their effectiveness is strongly strain-dependent and requires precise optimization of processing parameters to ensure stability and functionality across diverse food systems.

## 1 Introduction

The food and agriculture industries are rapidly changing, driven by innovation and emerging technologies to meet dynamic consumer preferences and sustainability demands. Consumers are becoming increasingly health-conscious, prioritizing functional foods that offer health benefits beyond basic nutrition. Functional foods may exert these effects either inherently or through enrichment during processing with bioactive compounds such as vitamins, trace elements, proteins, or probiotics (Welk et al., 2025). The growing industrial interest in probiotic-enriched foods has intensified research efforts to optimize processing technologies that preserve microbial viability and functionality.

Probiotics, defined as live microorganisms that improve health when consumed in sufficient quantities, primarily alter the gut microbiota, which plays a critical role in enhancing host health (Hill et al., 2014). Their beneficial effects are primarily associated with modulation of gut microbiota composition and metabolic activity, including enhanced short-chain fatty (SCFA) acid production, thereby contributing to improved intestinal homeostasis (de Assis et al., 2025; Duysburgh et al., 2025). To exert these effects, probiotics must remain viable at sufficient levels throughout storage and gastrointestinal transit. In food applications, products are typically formulated to deliver at least 10^6^ colony forming units (CFU)/g (or mL) at the end of shelf life, although the effective dose may vary depending on the specific strain and matrix (Hill et al., 2014). In food systems, probiotic microorganisms are predominantly represented by lactic acid bacteria (LAB), particularly species formerly classified within the genus *Lactobacillus* and now reclassified into genera such as *Lacticaseibacillus*, *Limosilactobacillus*, and *Lactiplantibacillus*, as well as members of *Bifidobacterium*. In addition, selected strains of *Streptococcus*, *Enterococcus*, and the yeast *Saccharomyces* are employed in specific functional or fermented applications (Marco et al., 2021).

Incorporating probiotics into food presents significant challenges, as exposure to acidic environments during storage and to gastric and intestinal conditions during digestion can substantially reduce cell viability (Sultana et al., 2023; Moreno et al., 2025). Addressing these challenges is critical for maintaining probiotic functionality and preserving product quality throughout storage and gastrointestinal transit. The food matrix and delivery format significantly influence probiotic survival and stability (Mahmoud et al., 2020). Consequently, increasing attention has been directed toward non-thermal processing technologies as alternative strategies to enhance probiotic performance (Demir et al., 2023; Vieira et al., 2024). Techniques such as ultrasound (US), high hydrostatic pressure (HHP), and pulsed electric fields (PEF) have been investigated as sub-lethal interventions capable of inducing adaptive stress responses, modulating membrane permeability, and stimulating metabolic activity, thereby enhancing stress tolerance and preserving viable cell counts under controlled conditions (Pankiewicz et al., 2020; Ma et al., 2023; Brito et al., 2024). This review critically examines the technological challenges associated with probiotic incorporation into foods, with particular emphasis on non-thermal approaches that aim not only to maintain viability but also to enhance functional and metabolic performance.

Although numerous experimental studies have investigated probiotic survival under specific stress conditions, including thermal exposure, acidic environments, and gastrointestinal simulation, the literature remains fragmented. Several reviews have addressed individual aspects of this field, such as non-thermal technologies, encapsulation strategies, or factors influencing probiotic survival (Demir et al., 2023; Pereira et al., 2018; Rajam and Subramanian, 2022; Vieira et al., 2024). However, these topics are often discussed independently, with limited comparative evaluation of how different technological approaches perform across diverse food matrices. In particular, the interaction between matrix-specific stress factors (e.g., pH, water activity, processing intensity) and optimized protective strategies remains insufficiently integrated within a single analytical framework (Afzaal et al., 2019; Hasgucmen and Sengun, 2020; Kemsawasd and Chaikham, 2020; Mahmoud et al., 2020). Moreover, emerging strategies, such as combining non-thermal technologies with encapsulation systems or incorporating postbiotics and paraprobiotics as complementary bioactive approaches, are typically treated separately rather than comparatively (Siciliano et al., 2021; Parvarei et al., 2021; McGillin et al., 2022). Therefore, a structured synthesis that comparatively evaluates technological interventions, biological responses, and matrix-dependent performance is needed to clarify current limitations and guide future optimization strategies.

This review aims to comparatively evaluate the effects of non-thermal processing technologies on probiotic viability, metabolic stability, and functional performance across diverse food matrices, while integrating emerging biological and regulatory considerations to identify current research gaps and future optimization strategies.

## 2 Methodology

This review was conducted as a structured critical narrative review rather than a formal systematic review or meta-analysis. A comprehensive literature search was performed using the electronic databases PubMed, Scopus, Web of Science, and Google Scholar to identify relevant peer-reviewed publications and authoritative guidance documents.

To ensure coherent thematic coverage, the search strategy was organized into five conceptual clusters: (i) probiotic viability and stability across food matrices, (ii) non-thermal and mild processing technologies influencing probiotic functionality, (iii) processing-driven functional and sensory outcomes, (iv) emerging biological/biotechnology-based approaches (e.g., paraprobiotics, postbiotics, bacteriophages where relevant to food applications), and (v) safety and regulatory considerations. Representative keyword combinations included: (i) (“probiotic” OR “probiotic foods”) AND (“sensory properties” OR “nutritional benefits” OR “antimicrobial activity” OR inflammation OR immunomodulation OR “cholesterol reduction” OR “lactose intolerance”), (ii) (“probiotic viability” OR “probiotic survival” OR “cell count” OR “log CFU”) AND (yogurt OR dairy OR meat OR frozen OR fruit OR “plant-based” OR bakery), (iii) (“probiotic”) AND (“non-thermal processing” OR “high hydrostatic pressure” OR HHP OR “pulsed electric field” OR PEF OR “cold plasma” OR “ultrasound” OR “ohmic heating”) AND (viability OR survival OR stability), (iv) (“bacteriophage” OR phage therapy), (v) (“postbiotic” OR “paraprobiotic”) AND (functional food OR viability OR stability), (vii) (regulation OR legislation OR safety OR EFSA OR FDA) AND (probiotic food).

Initial searches using representative combinations yielded several hundred records per query, e.g., >1,000 “probiotic viability” OR “probiotic survival” OR “cell count” OR “log CFU” AND yogurt OR dairy OR meat OR frozen OR fruit OR “plant-based” OR bakery.” After removing duplicates and excluding unrelated fields, non-food applications, non-English articles, and non-relevant domains (e.g., purely medical supplementation studies without food matrix context), records were screened by title and abstract.

Studies were included if they: (i) reported original experimental or regulatory data, (ii) addressed probiotic viability, functional stability, or safety in food systems, (iii) provided quantitative or mechanistic insights relevant to processing technologies. Screening was performed in two stages (title/abstract followed by full-text assessment), and the final synthesis prioritized original experimental studies for comparative discussion of strain/matrix, processing conditions, and viability outcomes.

In addition to relevance-based inclusion criteria, methodological rigor and scientific quality were also considered during the evaluation and synthesis of the selected studies. Preference was given to peer-reviewed studies with clearly described experimental designs, appropriate control conditions, adequate analytical methodologies, and statistically supported findings related to probiotic viability, functional stability, processing performance, and safety. Studies lacking sufficient methodological transparency or direct relevance to food-based probiotic applications were not prioritized in the comparative synthesis. However, as this work was designed as a structured critical narrative review rather than a formal systematic review, no standardized quantitative risk-of-bias assessment tool was applied.

The primary search window focused on recent developments (2015-2025/early 2026) to capture current non-thermal processing applications and emerging product concepts. However, earlier foundational, definitional, and internationally recognized consensus sources were intentionally retained where conceptually necessary (e.g., FAO/WHO et al., 2002; Taverniti and Guglielmetti, 2011; Hill et al., 2014/2015). These earlier references were included exclusively to support terminology, regulatory definitions, and baseline conceptual frameworks, which remain valid and are not time-dependent. Importantly, the experimental core of the review is predominantly based on contemporary research: the majority of included original studies were published after 2017, with more than 80% of the experimental evidence dating from 2018 onward. This ensures that the synthesis reflects recent technological advances in probiotic processing, non-thermal interventions, and stability enhancement strategies.

In the final reference set (*n* = 155), review-type publications account for approximately 9% of the total citations and were used selectively to (a) map terminology and state-of-the-art context, (b) support mechanistic interpretation of non-thermal technologies, and (c) contextualize regulatory frameworks. The primary evidence base of the manuscript is therefore composed of original experimental studies and authoritative guidance documents.

## 3 Probiotic foods

Dietary choices are increasingly recognized as important determinants of human health (Marco et al., 2021). Accordingly, the probiotic market has expanded in recent years, reflecting growing awareness of their potential health benefits. The current probiotic market includes fermented foods, beverages, dietary supplements, and formulated products. However, food-based delivery systems are often preferred because the food matrix can provide protective effects that enhance probiotic stability and functionality (Afzaal et al., 2019; Mahmoud et al., 2020). Probiotics can be successfully introduced into foods to enhance flavor, extend shelf life, and improve human health (Lee et al., 2017; Liu et al., 2024). Increased consumer demand for healthier foods has resulted in a diversification of probiotic products and their integration into various food matrices. A review of the literature reveals that, in addition to dairy products, fruits and vegetables are widely used in the production of probiotic foods, followed by cereal-based and meat products (Dimitrovski et al., 2021; Kokwar et al., 2022; Tukel and Sengun, 2024; Table 1). Fruits and vegetables naturally contain healthy nutrients and are free of allergens such as lactose and casein, making them an ideal matrix for introducing probiotics (de Oliveira et al., 2021). Many plant-based fermented foods have long been part of traditional dietary practices and represent important elements of cultural heritage (Marco et al., 2021). The production of fermented foods and beverages involves the controlled growth of naturally occurring microorganisms or the use of selected microbial populations and the action of microbial enzymes. High concentrations of viable microorganisms, particularly LAB, contribute to the accumulation of bioactive metabolites and enhancement of antioxidant activity, as demonstrated in recent studies reporting significant increases in phenolic and flavonoid contents following probiotic fermentation (Yuan et al., 2025; da Silva et al., 2023). As illustrated in Table 1, probiotic foods can be obtained by adding probiotics to the food as starter cultures, followed by a fermentation process that results in nutritional and quality improvements, changes in organoleptic profiles, enhanced safety and shelf life, peptide production, and antioxidants.

**Table 1:** Comparison of probiotic food types and production methods.

Probiotic strain	Inoculum	Form	Food type	Main ingredients	Method	Storage	References
*L. plantarum* LP115 400B *L. rhamnosus* LR32 200B *B. lactis* BB12	10^10^ CFU/g	Free	Fermented salami	Beef, fat, starter cultures, salt, nitrite salt, antioxidant, dextrose	Fermentation	4°C, 60 days	Tukel and Sengun (2024)
*L. rhamnosus* K3 *L. johnsonii* K4	10^9^ CFU/g	Free	Puree	Apple, chia seed, oat bran, and oat flakes	Fermentation	4°C, 24 h	Küçükgöz et al. (2024)
*L. casei* 01 *L. rhamnosus* ATCC 5310 *L. plantarum* 299v	10^8^ CFU/mL	Free	Beverage	Apple, pear, dried date, pea protein isolates, and *Spirulina platensis*	Fermentation	4°C, 90 days	Yahsi and Sengun (2024)
*L. plantarum 299v, B. animalis* Bo	10^7^ CFU/g	Free	Cheese	Skim milk, bigels, avocado oil, and coconut oil	Fermentation	4°C, 5 days	Machado et al. (2023)
*L. rhamnosus* LGG *L. casei* 43 *B. lactis* BB-12	10^9^ CFU/g	Free	Yogurt	Yogurt and mango peel powder	Fermentation	-	Zahid et al. (2023)
*L. plantarum* CECT 220	10^8^ CFU/mL	Free	Beverage	Carrot, orange, and inulin	Fermentation	4°C, 21 days	Valero-Cases et al. (2023)
*L. acidophilus* B103	10^9^ CFU/mL	Free	Beverage	Orange	Inoculation into the final product	4°C, 21 days	Kardooni et al. (2023)
*L. plantarum* NCIM 2084 NCIB 853	10^9^ CFU/mL	Free	Beverage	Oats, barley, buckwheat, and red rice	Fermentation	4°C, 10 days	Kokwar et al. (2022)
*L. acidophilus* NRRL B-4495 *L. plantarum* ATCC-1491	10^10^ CFU/g	Free and microencapsulated	Bread	Sorghum flour, salt, sugar, and instant yeast	Inoculation into the final product	4-7°C, 10 days	Ghasemi et al. (2022)
*L. casei* 431	10^7^ CFU/mL	Free	Beverage	Pumpkin	Fermentation	4-7°C, 10 days	Dimitrovski et al. (2021)
*L. rhamnosus* HN001	10^10^ CFU/g	Free	Cheesecake	Quark cheese, honey, and butter	Inoculation into the final product	20°C, 120 days and 4°C, 5 days	Hasgucmen and Sengun (2020)
*L. acidophilus* PTCC 1643 *B. bifidum* PTCC 1644	10^9^–10^10^ CFU/mL	Microencapsulated	Beverage	Grape	Inoculation into the final product	4°C, 8 weeks	Mokhtari et al. (2019)

Recent experimental evidence indicates that probiotic-enriched fermented foods may enhance gut microbial balance and metabolic outputs such as SCFA (Duysburgh et al., 2025; Prangthip and Chaikham, 2025). When probiotics are added during fermentation, non-probiotic starter cultures may be used to exert technological roles, optimizing the fermentation process depending on the specific properties of the product (Alinovi et al., 2025; Wu et al., 2025). Alternatively, selected probiotic strains, most commonly belonging to the genera *Lactobacillus* and *Bifidobacterium*, may be incorporated as adjunct cultures or directly added to food matrices to develop non-fermented or minimally fermented probiotic foods (Kardooni et al., 2023; Küçükgöz et al., 2024).

Developing probiotic food products is a complex process influenced by several factors, including the selection of an appropriate food matrix and probiotic strain, culture stability, microbial interactions, safety, sensory acceptance, and nutritional value. Ensuring the viability of probiotic populations throughout storage and consumption is a key issue in this sector. Probiotics, whether part of the fermentative population or added to food matrices as functional strains, must survive production and storage to exert their benefits. Maintaining adequate probiotic viability until consumption is widely recognized as essential for ensuring probiotic functionality (Hill et al., 2014). In recent years, emerging non-thermal technologies such as ultrasound, HHP, high-pressure homogenization (HPH), and PEF have been investigated as sub-lethal processing strategies for raw materials or directly for probiotic cultures (Demir et al., 2023; Vieira et al., 2024). Experimental studies indicate that controlled treatments may enhance stress tolerance, support metabolic activity, and improve probiotic stability, while maintaining or improving the overall quality of functional food products (Siroli et al., 2020; Pankiewicz et al., 2020; Ma et al., 2023).

The rapid expansion and diversification of the probiotic and/or fermented food products market highlight the need for updated and harmonized regulatory frameworks (Szajewska and Vinderola, 2024; Panel et al., 2022). A lack of global harmonization, along with differences in national approaches and regulatory frameworks, persists and has led to significant inconsistencies and misconceptions about probiotic products, their efficacy, and applications, as individual countries and agencies continue to apply distinct standards and regulatory criteria (ISAPP, 2022).

Producers of certain fermented foods may, on occasion, categorize or label their products as “probiotic foods” or “contains probiotics” to indicate the inclusion of living, health-promoting microorganisms. Nevertheless, the term “probiotic” should only be used for products demonstrated to provide health benefits due to the action of specific, identifiable, and adequately specified levels of live microorganisms (Hill et al., 2014). Therefore, the terms “fermented foods” and “probiotics” should not be used interchangeably. Probiotic foods are products “intended to maintain or enhance a healthy state in healthy or at-risk populations” and contain living microorganisms in accordance with the probiotic definition (Hill et al., 2014). Consequently, fermented foods cannot be automatically classified as probiotic foods unless strain-specific health benefits have been demonstrated under defined conditions of use. This has also been confirmed in a consensus statement, which aims to provide researchers, stakeholders, and consumers with a clear definition of fermented foods, differentiate between fermented foods and probiotics, summarize advances in knowledge on the microbial ecology of those foods, and characterize the health effects of fermented foods and their position in dietary guidelines (Marco et al., 2021).

Probiotics are classified into three regulatory categories: probiotic foods, probiotic dietary supplements, and live biotherapeutic products. The latter two are available as pills, capsules, or freeze-dried cell powders (Szajewska and Vinderola, 2024). These categories are reflected in corresponding regulations, depending on their intended use, health claims, safety, and quality control. They are also categorized by whether they aim to maintain consumers’ health by supporting normal physiological functions (probiotic foods and supplements) or preventing and treating diseases or symptoms (biotherapeutics). Dietary supplements and probiotic-containing food products generally do not assert specific health claims (Hill et al., 2014; Szajewska and Vinderola, 2024). Probiotic products intended to treat or prevent disease, and claiming therapeutic or diagnostic use, are categorized as “drugs” (medical or therapeutic products). These require extensive pre-marketing approval and are subject to regulation in accordance with relevant legislation (ISAPP, 2022; Szajewska and Vinderola, 2024).

Probiotics are generally regulated under food or drug legislation rather than under specific probiotic laws. However, regulatory definitions, admissible claims, and quality standards vary widely across countries, creating classification challenges and limiting global harmonization (Szajewska and Vinderola, 2024). Many regulatory systems rely on positive lists of microorganisms considered safe for use. In the EU, the Qualified Presumption of Safety (QPS) framework governs strains intentionally used in foods, while in the United States safety is established through the GRAS process (Panel et al., 2022). Although the QPS list supports market entry, the term “probiotic” in the EU is generally restricted on labels since not considered a health claim under the EU regulatory framework, unless approved by EFSA and with some exceptions and under specific conditions (Szajewska and Vinderola, 2024). To date, only specific yogurt cultures have received authorized health claims related to improved lactose digestion within the EU framework. In addition, most countries have enacted more or less restrictive rules on the use of claims related to probiotics and prebiotics. In any case, the presence of truthful and substantiated claims is fundamental: probiotic foods must be correctly labelled to protect the consumer from misleading information so that they can make informed choices. The label of a probiotic food must include the following information: the genus, species, and strain of the probiotic; the minimum viable number of each strain; the serving size; an accurate description of the clinically validated physiological effect of the strain; proper storage conditions; and the contact details of the company for consumer information and safety under the conditions of recommended use (ISAPP, 2022).

Consumer acceptance is strongly linked to transparency, trust, and education. Accurate labeling, alongside educational efforts by organizations and the research community, enhances consumer confidence by providing clear information, terminology, traceability, and evidence-based guidance (Fredua-Agyeman and Larbi, 2025). Robust, sustainability-based communication strategies that highlight scientific evidence empower consumer decision-making. While traditional probiotic foods use naturally occurring strains, understanding the molecular mechanisms behind probiotic benefits and advances in biotechnology could lead to improved strains through genetic modification. Engineering microbial strains is a promising tool for developing new microorganisms with enhanced functionalities, such as targeted metabolite production or improved gut colonization (Qiao et al., 2020). However, this approach raises ethical and safety concerns, including horizontal gene transfer, antimicrobial resistance, and uncontrolled colonization (Szajewska and Vinderola, 2024; Panel et al., 2022). Mitigation strategies, clear regulatory support, robust safety testing, and public communication can help establish an ethical framework based on transparent disclosure, which is indispensable for maintaining public trust. Similar ethical concerns arise regarding the use of bacteriophages as natural antimicrobials in probiotic foods to combat antibiotic resistance. Bacteriophages can target specific pathogens with minimal disruption to the host microbiome, but improvements in processing technologies and current regulatory frameworks are needed to balance innovation with precaution (Thompson et al., 2024; Wen et al., 2024).

### 3.1 Sensory properties of probiotic foods

Despite the increasing popularity of probiotic foods, technological challenges extend beyond maintaining cell viability during processing, storage, and gastrointestinal transit. Probiotic strains must also tolerate freeze-drying, oxidative and osmotic stress, and temperature fluctuations while preserving product quality (Gurram et al., 2021; Hao et al., 2021). Importantly, the incorporation of probiotics, either as starters, co-starters, or adjunct cultures, should not adversely affect sensory attributes, as these are critical determinants of overall consumer acceptance (Haro-González and Velásquez-Reyes, 2025). The sensory profile of fresh or fermented foods is usually defined by many volatile organic compounds belonging to various functional groups (acids, alcohols, ketones, esters, etc.) present in well-defined proportions. Probiotics, LAB, as well as *Bifidobacterium* spp. and certain yeast strains, have demonstrated the ability to modulate volatile compound production, particularly esters and alcohols, thereby influencing the aroma and sensory characteristics of fermented food matrices (Niamah et al., 2023). However, excessive acidification or uncontrolled metabolite production may lead to sourness, bitterness, or off-flavors. In dairy systems such as yogurt and cheese, probiotic and mixed starter cultures can modulate key aroma compounds, including acetaldehyde and diacetyl, thereby influencing volatile flavor profiles and overall product acceptability (Biçer et al., 2024; Ma et al., 2026). Similarly, in fermented sausages, probiotic starter cultures can modulate lipid oxidation and amino acid metabolism, thereby influencing volatile compound formation and contributing to flavor complexity (Liu et al., 2024). Plant-based matrices present additional challenges due to inherent beany or green notes. Probiotic fermentation can mitigate these sensory limitations by modulating volatile profiles, reducing off-flavor compounds, and degrading anti-nutritional factors such as phytates, thereby enhancing both palatability and nutritional value (de Sousa Melo et al., 2025; Alinovi et al., 2025). Nevertheless, excessive lactic acid production may compromise sensory balance. Optimizing probiotic metabolic performance necessitates targeted strain selection and process control to modulate aroma formation and enhance palatability. Furthermore, comprehensive sensory evaluation supported by advanced analytical techniques, including electronic nose, GC–MS profiling, metabolomics, and predictive modeling approaches, is essential for establishing robust correlations between volatile compounds and sensory perception, thereby providing reliable insights into consumer acceptance (Zhang et al., 2025; Zong et al., 2025).

Taken together, these findings emphasize the importance of maintaining probiotic viability for delivering health benefits, ensuring stable metabolic activity, and achieving consistent sensory quality in functional food systems.

### 3.2 Nutritional and health benefits of probiotic foods

Food digestion directly influences gastrointestinal tract (GIT) functionality, which plays a central role in metabolic regulation and immune homeostasis (Lee et al., 2017; Wu et al., 2021). Dietary patterns can modulate gut microbiota composition, thereby affecting the development of metabolic and inflammatory disorders. In this context, functional food components capable of surviving digestion and exerting physiological effects are essential elements of a balanced diet. Probiotics, when administered in adequate amounts, confer documented health benefits. Their mechanisms of action include adhesion to intestinal epithelial cells, reinforcement of epithelial barrier integrity, competitive exclusion of pathogens, modulation of immune responses, production of antimicrobial compounds, and regulation of host–microbiota interactions (Wu et al., 2021; Moreno et al., 2025). These mechanisms collectively contribute to improved gut homeostasis and metabolic balance. Probiotics are naturally present in several fermented foods, such as kefir, and they can be incorporated into foods. Most consumers prefer to incorporate probiotics into their diets through foods. Consumer preference tends to favor food-based delivery systems over pharmaceutical forms, highlighting the importance of developing probiotic foods that combine technological stability with functional efficacy (Welk et al., 2025). Accumulating experimental evidence indicates that probiotic consumption may influence metabolic outputs such as SCFA production, immune signaling pathways, and inflammatory markers, although strain-specific effects and dose dependency remain critical considerations (Duysburgh et al., 2025; Jiang et al., 2025).

#### 3.2.1 Antimicrobial activity of probiotic foods

Experimental studies have demonstrated that probiotic-fermented foods contain bioactive metabolites that exert antimicrobial activity based on the production or release of lactic acid, antimicrobial peptides (e.g., bacteriocins), hydrogen peroxide, etc. (Azizkhani et al., 2021; Bayram et al., 2024; da Silva et al., 2023). Probiotics can also limit pathogen invasiveness by competing with host cell receptors or by secreting antioxidants. Additionally, probiotics can exert their antimicrobial action by modifying the expression of genes responsible for the colonization of intestinal pathogens (Iglesias et al., 2017; Moreno et al., 2025). Despite the elucidation of mechanisms underlying probiotic–pathogen interactions in the GIT, the interactions between probiotics and pathogens in foods remain largely unexplored. Iglesias et al. (2017) reported that the co-inoculation of *L. monocytogenes* with *L. rhamnosus* GG reduced pathogen invasiveness due to competitive metabolite production and lowered pH. These findings suggest that probiotic-derived metabolites play a crucial role in suppressing foodborne pathogens.

#### 3.2.2 Antiproliferative effects of probiotics

Several experimental studies have demonstrated that probiotic strains and their derived metabolites exert antiproliferative effects against colorectal cancer in both *in vitro* and *in vivo* models, partly through the modulation of key signaling pathways such as BMP and β-catenin (Alan et al., 2025; Sepehr et al., 2024). Water-soluble peptide extracts derived from cow milk and buffalo cheddar cheese (produced by *L. lactis* and *L. cremoris*) demonstrated significant growth inhibition activity against the human lung (H-1299) cell line (Rafiq et al., 2018a). The extracts also showed efficacy against a range of cancer cell lines, including adenocarcinoma (HCT-116) and colon (HT-29) cell lines (Rafiq et al., 2018b). Rosa et al. (2020) investigated the *in vivo* antiproliferative effects of probiotic whey beverages containing *L. acidophilus* La-03, *L. acidophilus* La-05, *B. animalis* Bb-12, and *L. casei* 01. The authors suggested that whey beverage supplemented with *L. casei*-01 may exhibit potential antiproliferative activity against prostate cancer cells under *in vitro* conditions. Mostafa et al. (2020) investigated the antiproliferative effect of probiotic date juice against Caco-2 and Hep-2 cell lines. They discovered that probiotic date juice had anticancer activity against Hep-2 but not against Caco-2. Collectively, these findings indicate that probiotic-derived metabolites may contribute to the modulation of cancer-related cellular pathways, although strain specificity, dosage, and matrix effects remain critical variables.

#### 3.2.3 The role of probiotics in inflammation

Inflammation represents a biological response to internal or external stressors, including infection and tissue injury, and plays a role in maintaining physiological homeostasis (Wu et al., 2021). Gut inflammation is accompanied by increased intestinal epithelial permeability, which enables pathogens to enter the bloodstream and subsequently migrate to other organs (Jiang et al., 2022). The equilibrium of the gut microbiota influences physiological processes, including digestion, absorption, detoxification and immunity. Historically, antibiotics have been a mainstay for treating intestinal inflammation. However, their excessive use can lead to the formation of antibiotic-resistant pathogens, reduce their efficacy, and disrupt the delicate balance of the gut microbiota (Szajewska and Vinderola, 2024). In contrast, selected probiotic strains have been investigated as supportive strategies with a more favorable safety profile compared to long-term antibiotic use. These advantages offer substantial opportunity for the prevention and improvement of intestinal inflammation. Probiotics exert anti-inflammatory effects by modulating immune responses, reinforcing epithelial barrier integrity, and altering inflammatory cytokine profiles (Sepehr et al., 2024; Jiang et al., 2025). For instance, multi-strain probiotic administration has been shown to enhance tight junction integrity, increase SCFA production, and reduce pro-inflammatory mediators in advanced gut disease models (Maxan et al., 2026).

#### 3.2.4 Immunomodulation

Gut microbiota has recently been the subject of intensive research owing to its critical role in host health. The ingestion of probiotics can influence and regulate the host immune system. According to Wu et al. (2021), probiotic-fermented raspberry juice (*L. casei*) altered the gut microbiota to achieve a healthy *in vitro* state, which was marked by an increase in the production of valeric and isovaleric acids. In mice with no underlying health issues, all fermented raspberry juice treatments also improved the production of butyric, acetic, and isovaleric acids. There was also a rise in the expression of certain genes, including *Claudin-1*, *ZO-1*, *Claudin-4*, *OCLN*, *E-cadherin*, and *Muc-2*. Harahap et al. (2023) reported that probiotic candidates derived from traditional Sumatran fermented products exert immunomodulatory effects in murine models.

#### 3.2.5 Effects on blood cholesterol

Hypercholesterolemia is a significant factor contributing to the development of primary hypertension. Clinical and experimental studies have reported reductions in serum cholesterol levels following probiotic consumption, although strain- and population-specific responses have been observed. This result may be associated with the interaction between gut microbiota and host lipid metabolism, since cholesterol homeostasis is largely regulated through hepatic synthesis, intestinal absorption, and microbial bile salt metabolism. Probiotics have been evidenced in certain trials to facilitate a decline in blood cholesterol levels and increase in low-density lipoprotein’s resistance to oxidation, which can result in a lessening in blood pressure. Fan et al. (2024) investigated probiotic sea buckthorn (*Hippophae rhamnoides* L.) juice for its cholesterol-lowering potential and found that the fermentation of sea buckthorn juice by *L. plantarum* 10,211 may boost the lipid-lowering activity of sea buckthorn. In another study, Papakonstantinou et al. (2024) investigated the effects of probiotics (*L. casei* Shirota and *L. rhamnosus* GG) and orange juice fortified with vitamin D3 (2000 IU) on cardiometabolic risk factors in overweight and obese adults following a Westernized diet. The findings indicated that the group consuming orange juice combined with vitamin D3 and probiotics had statistically significantly decreased low-density lipoprotein cholesterol (LDL-C) levels than the control group. These findings suggest that probiotic-enriched foods may play a role in personalized nutrition strategies aimed at reducing metabolic risk and improving lipid profiles.

#### 3.2.6 Effects on lactose intolerance

Lactose intolerance is a genetically determined deficiency of β-galactosidase (lactase), an enzyme responsible for hydrolyzing lactose into glucose and galactose. Lactase activity in mammals is highest during lactation, then declines after weaning, causing a decrease in lactose absorption. Non-absorbed lactose in the small intestine is transported to the colon, where it is metabolized by the microbiota, resulting in the production of SCFAs and gases, predominantly hydrogen (H2), carbon dioxide (CO2), and methane (CH4), which contribute to gastrointestinal symptoms (Hill et al., 2014). For instance, after consuming dairy products, lactose-intolerant individuals experience gas, diarrhea, and abdominal pain. *L. bulgaricus* and *B. bifidum* are two main strains that can synthesize the β-galactosidase enzyme, which can hydrolyze lactose in dairy products and increase tolerance. Masoumi et al. (2021) studied the impact of yogurt prepared using *L. acidophilus* and *Bifidobacterium* spp. on patients with lactose intolerance. Their findings revealed that probiotic yogurt can safely and effectively lessen lactose intolerance symptoms.

## 4 Probiotic viability

### 4.1 Significance of probiotic viability

Probiotics are described as “live microorganisms that, when administered in adequate quantities, confer a health benefit on the host” (Hill et al., 2014). This definition underscores the significance of probiotic viability in achieving health effects. Regardless of the delivery method, probiotics must remain intact through the upper gastrointestinal tract to ensure health-promoting effects once they reach their site of action, according to guidelines from the World Health Organization (WHO) and the Food and Agriculture Organization of the United Nations (FAO) (FAO/WHO et al., 2002).

In food applications, maintaining sufficient viable probiotic populations throughout shelf life is essential to ensure functional efficacy (Hill et al., 2014). Effective daily intake levels are typically reported in the range of 10^8^–10^9^ CFU per day, depending on the specific strain, target population, and intended health outcome. Achieving and maintaining these levels requires careful consideration of formulation strategies, storage stability, and gastrointestinal survival.

Considering the information above, the viability of probiotics is typically regarded as a fundamental prerequisite for their functionality in promoting consumer health. Qayyum et al. (2024) stated that when LAB is used in functional foods or dietary supplements for cholesterol reduction, live viable cells may offer greater efficacy in cholesterol sequestering than non-viable or dead cells. Galdeano and Perdigon (2004) conducted a study in mice to investigate the effects of viable and non-viable *Lactobacillus* spp. on gut and mucosal immune stimulation. They demonstrated that viability is a prerequisite for the stimulation of the gastrointestinal immune system. A more recent review by Liu et al. (2022) similarly emphasized the role of viable probiotics in modulating immune signaling pathways, cytokine production, and intestinal immune homeostasis through interactions with host epithelial and immune cells. Conversely, some reports indicate functionalities associated with both viable and non-viable probiotics. This suggests that in some studies, viability is not a prerequisite for all probiotic effects, as not all cell mechanisms are directly linked to viability. In fact, dead cells have been shown to provide beneficial effects to consumers (Chung et al., 2019; Kang et al., 2021; Ocampo-Zaragoza et al., 2026). However, a product must have the designated probiotics in sufficient quantity at the moment of intake to be considered a probiotic food. Maintaining the strains’ metabolic activity and viability during manufacturing and storage, and eventual survival in the GIT, is essential. It is also crucial to maintain consumer confidence in probiotic products.

Several techniques have been proposed to enhance the viability of probiotics in diverse food products throughout their production and consumption (Gurram et al., 2021; Rajam and Subramanian, 2022). This is because probiotics are subjected to a wide range of stress factors, including fluctuations in temperature, acidity, and bile, high concentrations of specific ions or nutrient depletion, exposure to osmotic and oxidative stress within product matrices, and passage through the GIT. Such factors can detrimentally affect the viability and functionality of probiotics (Hao et al., 2021; Gurram et al., 2021). Probiotic survival during processing and storage is influenced by intrinsic product parameters such as pH, titratable acidity, oxygen availability, water activity, salt and sugar concentration, as well as processing conditions including fermentation parameters, heat treatment, cooling, packaging systems, and scale of production (Siroli et al., 2020; Ghasemi et al., 2022). After oral intake, probiotics transit through the gastrointestinal tract (GIT), which includes the mouth, stomach, small intestine, and colon. The GIT contains a range of physicochemical factors (bile acids, gastric acid, digestive enzymes) that impact the viability of probiotics (Afzaal et al., 2019; Moreno et al., 2025).

### 4.2 Stress factors affecting probiotic viability

**Figure 1:**
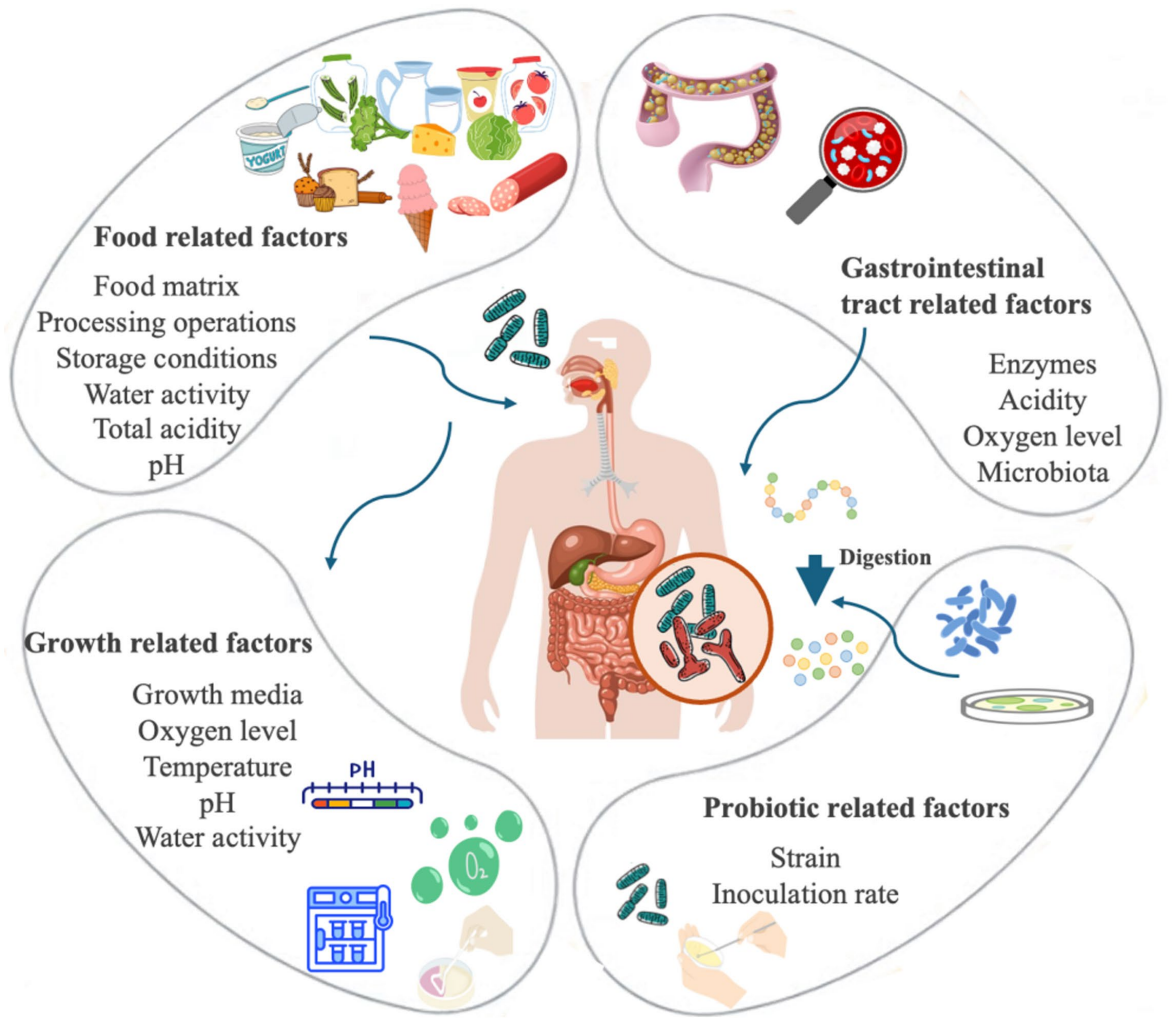
Factors influencing the viability of probiotics. The classification framework in this figure was developed by the authors based on a synthesis of the reviewed literature.

The factors affecting probiotic viability can be broadly classified into four categories: those related to the food, growth conditions, gastrointestinal system, and probiotic strain (Gurram et al., 2021; Figure 1).

The viability of probiotics can be influenced by food components or growth media such as prebiotic compounds and additives (sugars, salt, antimicrobials, flavorings, and even bacteriocins), which can exert a positive or negative effect (Ghasemi et al., 2022). Oxygen levels and redox potential are other significant factors, particularly during production and storage. The impact of oxygen on cell viability varies considerably among different genera. For example, *Lactobacillus* spp. exhibit greater tolerance to oxygen than *Bifidobacterium* spp., although oxygen exposure may still reduce viability depending on strain-specific characteristics. It is therefore recommended that oxygen concentration and packaging permeability be kept low to effectively control probiotic viability. Fermentation temperature also affects their viability. Most LAB exhibit optimal growth within a temperature range of 30–42 °C (Hao et al., 2021). Additionally, titratable acidity and low pH can affect probiotic viability during production and storage. Typically, fruit juices with low pH and high organic acid content exert major stress on probiotics. Among processing parameters, heat treatment is the most significant factor influencing probiotic viability, constraining their use in heat-treated foods. Moreover, the resilience of probiotics to heat stress can be enhanced through mild heat treatment before use. Applying non-lethal heat shock enables them to withstand a second, more intense heat stress, and heat adaptation enhances the thermal tolerance of *Lactobacillus* spp. (Bommasamudram et al., 2022; Hao et al., 2021). In frozen products, probiotic survival may be affected by ice crystal formation and osmotic shifts that disrupt membrane integrity. Cold-induced stress responses, membrane phase transitions, and osmotic flux have been identified as key determinants of survival in frozen and refrigerated systems (Kemsawasd and Chaikham, 2020; Shi et al., 2024). Table 2 presents potential solutions for factors influencing probiotic viability.

**Table 2:** Potential solutions regarding factors influencing probiotic viability.

Main factors	Detailed factors affecting viability	Suggested solution
Food matrix and formulation	Nutrients, pH, acidity, oxygen, microflora, osmotic pressure, and water activity	Stabilizer and protective carriers, growth promoter addition (e.g., prebiotics), strain adaptation (pretreating), microencapsulation
Dosage	Inoculation rate	Must be >10^6^ CFU/g-mL, prebiotic addition
Strain characteristics	Oxygen tolerance, heat tolerance, acid tolerance, and genetic stability	Use of resistant strains, appropriate strain selection
Processing conditions	Temperature, oxygen, humidity, heat treatment, packaging, freezing, and thawing	Glass packaging, low temperature, low humidity, microencapsulation, heat resistant strains, non-thermal techniques, strain adaptation (pretreating), prebiotic or cryoprotectant addition
Storage conditions	Temperature, pH, oxygen, and humidity	Suitable packaging, microencapsulation
Gastrointestinal system	Acid and bile salts, digestive enzymes, oxygen levels, and microbiota	Microencapsulation, growth promoters addition (e.g., prebiotic)

Probiotics are present in many foods, but maintaining appropriate levels during processing, storage, and transit through the GIT is challenging. This is important because low doses at the time of consumption will not be effective.

Considering the aforementioned factors that impact probiotic viability, the following section summarizes specific factors affecting probiotic viability in various food groups.

### 4.3 Viability of probiotics in various food products

The global probiotic food market has shown steady expansion, driven by increasing consumer demand for functional and health-oriented food products (Welk et al., 2025). From a technological standpoint, a wide range of food matrices have been investigated as potential delivery systems for probiotic microorganisms, provided that sufficient viability and functional stability can be maintained throughout processing and storage (Gurram et al., 2021).

Probiotics may be incorporated either during production as starter or adjunct cultures, or added after processing as functional ingredients (Rajam and Subramanian, 2022). They are commonly applied in dairy products such as yogurt, cheese, fermented milk, and ice cream (Biçer et al., 2024), as well as in baked goods (Zhang et al., 2018), meat products (Tukel and Sengun, 2024), and fruit- and vegetable-based systems (de Oliveira et al., 2021; Kardooni et al., 2023). However, incorporating probiotics into foods represents a significant scientific and technical challenge. One of the primary concerns is the viability and stability of these microorganisms. Probiotic survival within food matrices is strongly influenced by intrinsic factors (pH, water activity, redox potential, oxygen availability) and extrinsic processing conditions (heat treatment, mechanical stress, storage temperature), all of which may compromise membrane integrity, metabolic activity, and ultimately cell viability. Furthermore, heat treatment and other food manufacturing processes (e.g., mechanical) may influence probiotic viability.

#### 4.3.1 Probiotic viability in yogurt

The buffering capacity of milk ensures the survival of probiotics during fermentation and refrigeration, making dairy products the primary category of probiotic foods available on the market. Dairy matrices typically provide an environment and nutrients that probiotics can utilize for growth and viability. The dairy category includes products such as yogurt, fermented milk beverages, and frozen dairy desserts. Yogurt has been the most widely utilized delivery vehicle for probiotics to consumers (Biçer et al., 2024; Chaikham et al., 2025). Maintaining the recommended minimum viable probiotic level throughout storage and at the time of consumption remains essential for probiotic yogurt functionality (Hill et al., 2014). It is therefore crucial to maintain the optimal functionality of the probiotic throughout processing, storage, and transit through the GIT.

##### 4.3.1.1 pH and acidity

The metabolic characteristics and viability of probiotics are markedly influenced by pH and titratable acidity. Experimental evidence indicates that probiotic growth is strongly strain-dependent and influenced by environmental pH. For instance, *B*. *lactis* Bb12 has demonstrated active growth within a pH range of 5.3–6.7 under controlled conditions (Zhang et al., 2024), while optimal growth of *L. plantarum* strains has been reported near neutral pH values (6.5–7.0), with reduced growth observed at lower pH levels (Gökmen et al., 2024). Acidification during fermentation is one of the main factors limiting probiotic survival. Organic acids in their non-ionized form may diffuse across cell membranes and disrupt intracellular pH homeostasis, impairing viability (Călinoiu et al., 2016; Afzaal et al., 2019).

##### 4.3.1.2 Oxygen

Oxygen exposure during mixing, homogenization, and storage can promote reactive oxygen species formation, reducing probiotic viability, particularly in oxygen-sensitive genera such as *Bifidobacterium* (Gurram et al., 2021). The selection of appropriate packaging materials containing oxygen barriers and oxygen scavengers (also known as active and intelligent packaging) may also have a beneficial effect on the survival of probiotics. Probiotics can be maintained using other technological options, such as the addition of chemical materials (i.e., nitrogen), antioxidants (i.e., ascorbate and cysteine), or enzymatic treatments (i.e., glucose oxidase).

##### 4.3.1.3 Fermentation and storage

Probiotic cell counts may decline during active fermentation and subsequent refrigerated storage due to cumulative thermal, acid, and oxidative stresses. Fermentation temperature significantly affects probiotic survival and the sensory characteristics of probiotic yogurt. Many dairy-adapted probiotic strains exhibit optimal growth between 35–42 °C, although strain-specific differences are considerable. For example, certain *L. acidophilus* strains can grow at 40–42 °C (Wu et al., 2025). Temperatures exceeding 45 °C detrimentally impact probiotic growth and viability at all manufacturing stages (Călinoiu et al., 2016). Consequently, the fermentation process must be shorter at high temperatures to maintain probiotic counts. Probiotics should be added to the medium during the downstream stages of yogurt manufacturing, following pasteurization. Prior to use, moderate heat treatment can improve the heat tolerance of probiotics. Such adaptive responses may improve survival during fermentation and early storage; however, the magnitude of this protective effect remains strain-dependent. During refrigerated storage, probiotic decline is often associated with ongoing post-acidification, residual oxygen exposure, and gradual metabolic exhaustion. Although low temperatures slow metabolic activity, they do not fully prevent viability loss, highlighting the importance of controlling fermentation endpoint, oxygen diffusion, and storage conditions to maintain functional cell counts throughout shelf life.

##### 4.3.1.4 Inoculation rate and strain

Probiotic viability during fermentation and subsequent cold storage is also influenced by the initial inoculation level. While higher inoculum concentrations may accelerate fermentation kinetics and shorten production time, excessive cell density can intensify nutrient competition and accelerate acid accumulation, thereby increasing metabolic stress and promoting earlier entry into the stationary phase. Such conditions may ultimately compromise long-term viability during storage. Probiotic viability in yogurt also depends on the strain. It is necessary to select suitable probiotic strains, especially those that are acid, bile, and oxygen-tolerant and that can withstand the harsh environment of the GIT and products to ensure their health benefits.

##### 4.3.1.5 Homogenization and matrix composition

Mechanical processing may exert shear stress; however, optimized homogenization can improve cell dispersion. Matrix composition, including fat and lactose content, may further modulate probiotic survival by influencing buffering capacity and oxygen diffusion (Hasgucmen and Sengun, 2020; de Oliveira et al., 2021).

#### 4.3.2 Probiotic viability in meat products

Fermented and processed meat products represent a technologically challenging yet promising matrix for probiotic incorporation (Tukel and Sengun, 2024; Goktas et al., 2022). Unlike dairy systems, meat matrices are characterized by high protein and lipid content, limited carbohydrate availability, elevated sodium chloride concentrations, and reduced water activity (a_w_), all of which influence probiotic survival.

Salt concentrations commonly used in dry-fermented sausages (2–3% or higher) can exert osmotic stress, leading to membrane dehydration and reduced metabolic activity (Hao et al., 2021). Moreover, nitrites and nitrates, while essential for product safety and color development, may affect certain probiotic strains depending on their tolerance mechanisms. Competitive interactions with the native meat microbiota further complicate probiotic survival, as naturally occurring bacteria may produce bacteriocins, hydrogen peroxide, and organic acids that inhibit probiotic growth. Strain selection is therefore critical when developing probiotic meat products. Strains adapted to high-salt and low-a_w_ environments, particularly those originating from fermented meat ecosystems, may exhibit superior technological robustness. Encapsulation strategies, including alginate-based microencapsulation, have been investigated to improve probiotic stability under these harsh processing conditions. Additionally, synbiotic approaches combining probiotics with prebiotic substrates may enhance survival and functional performance within meat matrices (Mahmoud et al., 2020). Overall, probiotic viability in meat products is determined by a complex interplay between matrix composition, fermentation parameters, curing agents, and microbial ecology, necessitating strain-specific and process-optimized strategies.

#### 4.3.3 Probiotic viability in frozen products

Frozen dairy products such as ice cream and frozen desserts represent attractive vehicles for probiotic delivery due to their widespread consumption and relatively stable storage conditions. However, freezing imposes unique physicochemical stresses that may compromise probiotic viability. The primary mechanism of viability loss during freezing is associated with ice crystal formation. The formation of ice crystals during the freezing process can exert mechanical stress on cells, potentially leading to damage to the cell wall or rupture of the cell membrane (Goktas et al., 2022). In addition to the mechanical stress that can occur during the mixing stage for oxygen incorporation, exposure to oxygen can generate a toxic action due to the formation of reactive oxygen species in the cell (such as hydrogen peroxide, hydroxyl radicals, superoxide anions), resulting in cell death (Kemsawasd and Chaikham, 2020).

The freezing process can result in cold injuries caused by temperature reduction (temperature shock), condensation of solutes in the extracellular and intracellular medium, and cellular dehydration. Moreover, additional characteristics can influence the viability of probiotics within this matrix. These include the types of ingredients used, particularly the sugar content, which can impact the osmotic effects of this matrix on microorganisms, pH, and titratable acidity (especially in frozen variations). The aforementioned factors include storage time and temperature, as well as temperature fluctuations and abuse conditions, partial melting and re-freezing processes, and other related variables.

Considering the aforementioned factors, microencapsulation can be reasonably considered as a viable means of preserving cells over an extended storage period. However, the most appropriate packaging material must be selected to prevent the negative effects of oxygen. It is also necessary to ensure that the selected strain is appropriate for the product since different strains have varying degrees of resistance to external factors. The addition of prebiotics or cryoprotectants is also a promising strategy for probiotic protection.

#### 4.3.4 Probiotic viability in fruit and plant-based products

The use of fruit and plant-based foods as potential vehicles for probiotic foods is being increasingly investigated. Their vitamin, mineral, dietary fiber, and antioxidant content make them suitable substrates for probiotic growth. Conversely, using probiotics within fruit and vegetable matrices presents several challenges, largely owing to compounds that can inhibit probiotics during manufacturing and storage. Plant matrices also have unfavorable pH, anti-nutritional factors, and a lack of buffering capacity. Probiotic viability is therefore inextricably linked to the intrinsic characteristics of the food matrix and their interactions. Olivares et al. (2019) stated that incorporating considerable quantities of dissolved oxygen into fruit juices presents a significant challenge in maintaining probiotic viability. Since anaerobic and microaerophilic probiotic bacteria are the most widely used, it is paramount to recognize that high oxygen levels within the product may precipitate toxicity and viability loss. As an oxygen scavenger, vitamin C may therefore provide protection during storage and foster a more suitable anaerobic environment (which is not seen during fermentation), as stated by Olivares et al. (2019). A range of formulations combining mango juice with carrots, as well as various *Lactobacillus* spp., were evaluated in consideration of matrix composition (de Oliveira et al., 2021). The study found that the juice with a higher proportion of carrot pulp exhibited higher microbial viability, which can be attributed to its high fiber content. According to the scientists, dietary fibers may significantly affect probiotics’ ability to survive digestion and storage. It is crucial to highlight that phenolic compounds are essential for the viability of probiotics. For example, Valero-Cases et al. (2017) found that the metabolism of most phenolic compounds in pomegranate juice was associated with the proliferation of probiotics in the juice, with the degree of this association variable depending on the strain utilized. Probiotic viability is further impacted by heat treatment, incubation temperature, cooling, packing type, material and storage techniques, and production scale.

As demonstrated in the preceding studies, probiotic survival was attributable to the antagonistic and synergistic effects of multiple factors. For example, a reduction in pH can have a detrimental impact on cell viability, whereas the presence of dietary fiber and protein can protect cells from such stress.

#### 4.3.5 Probiotic viability in bakery products

Cereals constitute a major component of the human diet and provide carbohydrates, proteins, dietary fiber, vitamins, and minerals. Certain cereal-derived components may act as prebiotic substrates, promoting the growth of beneficial microorganisms such as *Lactobacillus* and *Bifidobacterium* spp. (Gibson and Roberfroid, 1995).

The development of probiotic food products within the bakery industry represents an innovative approach to expanding the non-dairy probiotic food market (Kokwar et al., 2022; Valero-Cases et al., 2023). Probiotic cereal-based products serve as alternatives to conventional probiotic dairy products, eliminating the need for refrigeration during storage and distribution. The food matrix of bakery products contains prebiotics that serve as a source of energy to facilitate probiotic growth. In contrast, the greatest challenge in developing a probiotic cereal-based product is maintaining probiotic viability because the production process involves high temperatures. Studies evaluating the incorporation of free probiotic cells into bread dough prior to baking have demonstrated significant reductions in viable counts, often below the minimum threshold required for probiotic functionality (Zhang et al., 2018; Hao et al., 2021). In addition to thermal stress, low water activity (a_w_) during storage may further reduce viability (Mahmud et al., 2022). To overcome these limitations, several strategies have been proposed, including microencapsulation techniques to enhance thermal protection and post-baking incorporation approaches to preserve viable cell counts (Pandey et al., 2021; Mis-Solval et al., 2019). Such technological interventions are essential to ensure that probiotic levels remain above the minimum effective threshold at the time of consumption.

### 4.4 Standardization of viability assessment

#### 4.4.1 Limitations of current methods

The most widely applied method for probiotic enumeration is plate count, requested by authorities and supported by ISO standards. Plate counts are inexpensive, straightforward, and allow visualization of colony morphology and contamination. However, they rely only on the ability of cells to replicate.

Cells that are metabolically active but unable to form colonies under standard laboratory conditions may therefore be underestimated. Consequently, CFU counts do not fully reflect the physiological state or functional potential of probiotic populations. Additional drawbacks include labor-intensive preparation, long incubation periods (typically 48–72 h), cell clumping effects, and inter-laboratory variability. Comparative studies have demonstrated discrepancies between culture-based enumeration and molecular quantification approaches, highlighting the limitations of CFU data alone in assessing probiotic viability (Hansen et al., 2020; Kiefer et al., 2020; Sun et al., 2024).

#### 4.4.2 Emerging tools

To overcome these drawbacks, several culture-independent techniques (e.g., flow cytometry, fluorescence *in situ* hybridization, nucleic acid amplification, bioluminescence, and biosensor technologies) have been introduced. Owing to their higher sensitivity, specificity, and efficiency, these tools are increasingly replacing conventional culture-based methods. Flow cytometry (FC), often combined with fluorescent dyes (e.g., PI/SYTO), enables rapid high-throughput assessment of membrane integrity, metabolic activity, and population heterogeneity; fluorescence-activated cell sorting (FACS) can further isolate subpopulations. Molecular methods such as qPCR and dPCR provide strain-level specificity and, when combined with viability dyes like PMA/EMA, can selectively detect DNA from intact cells (Kiefer et al., 2020). Multiplex PCR amplifies multiple targets simultaneously, offering rapid and sensitive detection of several species in a single reaction, though limited by PCR inhibitors, primer design, and the need for specialized equipment (Hansen et al., 2020). Spectroscopy and imaging techniques exploit microbial spectral signatures to distinguish and quantify strains even in mixed cultures, offering rapid and non-destructive analysis, but requiring costly instrumentation and technical expertise. Biosensor-based approaches use biological recognition elements (e.g., enzymes, antibodies, aptamers) to generate measurable optical or electrochemical signals. They provide high sensitivity, rapid detection, and real-time monitoring with potential for miniaturization, though challenges remain in stability, sample matrix effects, and high development costs.

Collectively, these methods expand the concept of “viability” beyond culturability, but their routine application demands careful optimization, skilled operators, and often significant investment (Sun et al., 2024).

#### 4.4.3 Reporting guidelines

Despite significant methodological advances, there is currently no universally harmonized operational definition of probiotic viability across research and regulatory contexts. While the probiotic definition emphasizes the presence of live microorganisms in adequate amounts (Hill et al., 2014), enumeration approaches vary substantially between studies. Some investigations define viability strictly as culturability (CFU), whereas others incorporate complementary indicators such as membrane integrity or molecular detection of intact cells. This inconsistency hinders inter-laboratory comparisons and regulatory harmonization. Harmonized guidelines are needed, including: (i) Reporting both CFU counts and complementary viability markers, (ii) Clear descriptions of protocols, media, and strain-specific conditions, (iii) Standardized combinations of culture-dependent and independent methods to capture replication, metabolic activity, and stress tolerance.

Such harmonization would improve comparability between laboratories, provide more reliable data for regulatory purposes, and strengthen consumer confidence in probiotic products (Sun et al., 2024).

## 5 Non-thermal innovative technologies used for enhancing viability

The food matrix and harsh food processing, storage, and post-ingestion conditions are examples of exogenous and endogenous factors that can potentially act as sub-lethal stressors of probiotic cells, making it difficult to maintain the biological activity of probiotics in relation to the methods used in the food industry.

**Figure 2:**
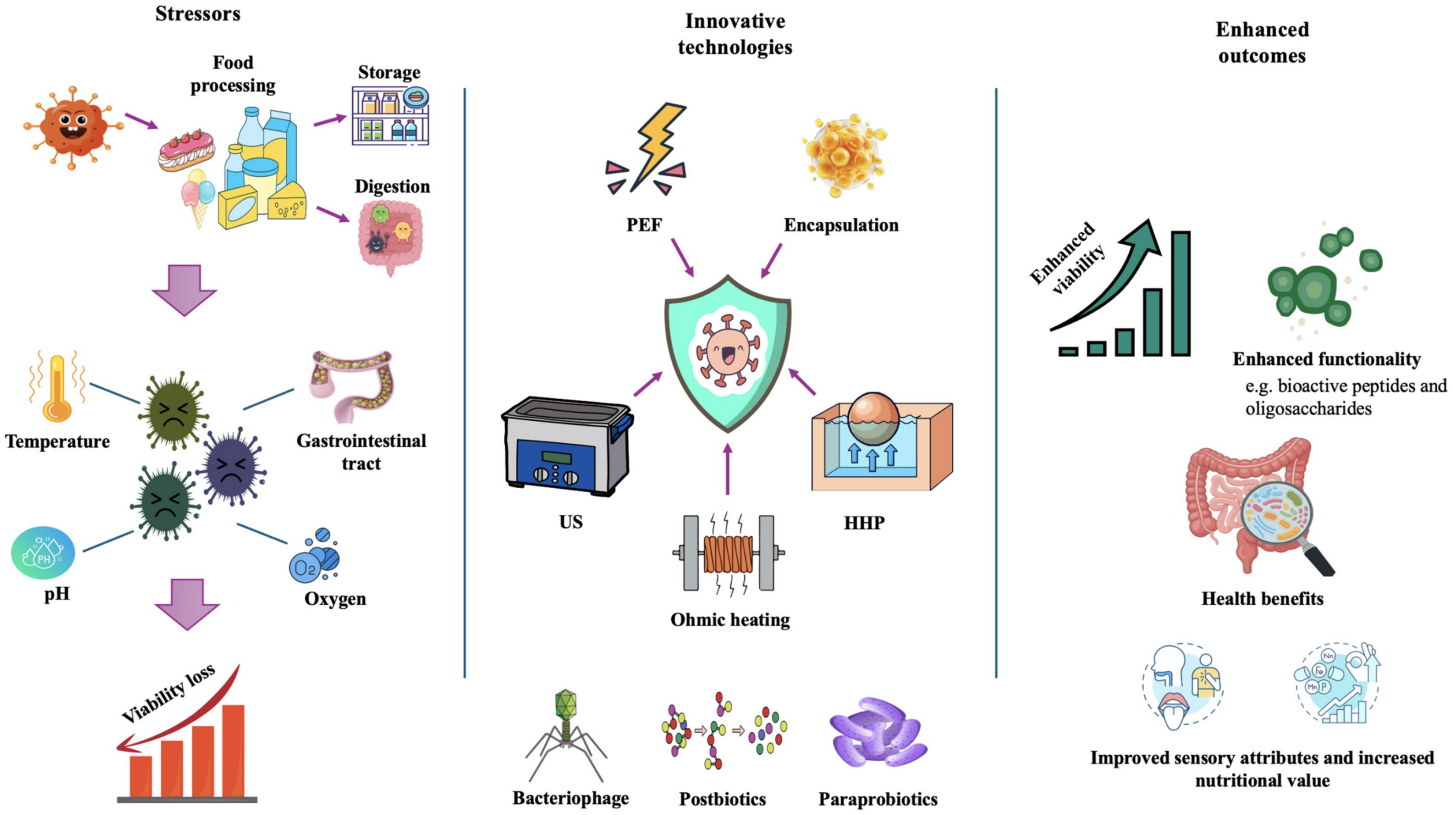
Innovative technologies and their impact on probiotic viability and functionality.

**Figure 3:**
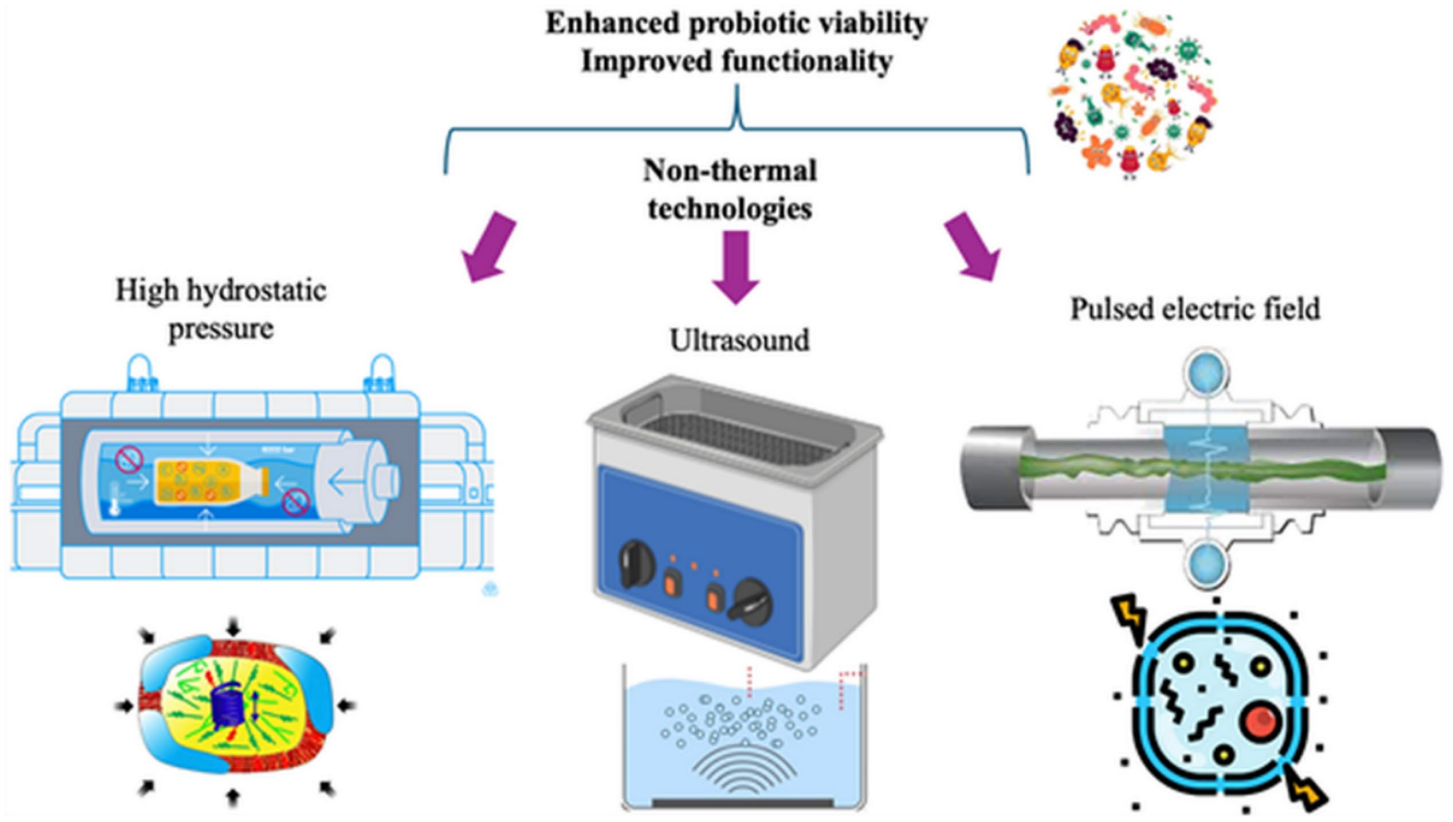
Schematic illustration of the mechanism of non-thermal technologies.

Non-thermal technologies such as US, HHP, and PEF could be used in food production without significantly affecting the viability of probiotic cells or even increasing their viability (Figures 2, 3). Table 3 shows the results of studies on the effects of non-thermal technologies on the viability of probiotics and the functional properties of foods.

**Table 3:** Studies on the effects of non-thermal technologies on probiotic viability and functional properties of foods.

Non-thermal techniques	Probiotic strain	Food matrix/medium	Results		References
			Viability	Functionality	
HPP (100–300 MPa, 10 min)	*L. casei* 01	Sweet corn milk yogurt	100 MPa treatment maintained probiotic viability above 7 log CFU/g during 6 weeks of refrigerated storage	100 MPa preserved physicochemical stability and enhanced gastrointestinal tolerance, whereas ≥200 MPa negatively affected texture and probiotic survival	Chaikham et al. (2025)
Low-intensity PEF (1–3 kV/cm, 50 pulses, 4 Hz)	*S. thermophilus and L. bulgaricus*	Yogurt	Enhanced LAB metabolic activity and accelerated fermentation kinetics under optimized PEF conditions	Fermentation time was reduced by up to 0.52 h, while lactic acid concentration increased by 76–90% without negatively affecting physicochemical or sensory properties	Miranda-Mejía et al. (2024)
Ultrasound (140, 370 and 440 W; energy density: 1600, 640 and 200 J/mL; 1 and 3 min)	*L. acidophilus* LA5	Fermented milk	Treatment with probe at 640 J/mL maintained LA5 viability	-	Brito et al. (2024)
Ultrasound (750 W, 2.5, 5, 7.5 and 10 min)	*L. casei* LC01	Strawberry-flavored probiotic fermented drinks	Processing for 2.5 min is better for probiotics, which survive well in the gut	Treatment for 2.5 min increased antioxidant activity	Mizuta et al. (2023)
Ultrasound (23 kHz, 150 W 3–5 min)	*L. brevis* LMG 6906	Fermentation medium	Sonication increased the viability of probiotics up to 1.19 logs depending on the parameters	γ-aminobutyric acid yield increased by 0.26 g/L after moderate ultrasound treatment	Shokri et al. (2021)
Ultrasound (20 kHz, 58.3 and 93.6 W/L, 3 s)	*L. plantarum* BNCC337796	Fermented apple juice	Sonication during lag and logarithmic phases promoted the growth and intensified the biotransformation of malic acid to lactic acid	Ultrasound positively affected the hydrolysis of chlorogenic acid to caffeic acid, the transformation of procyanidin B2 and decarboxylation of gallic acid	Wang et al. (2021)
Ultrasound (24 kHz, 300 W, 30 min) – microwave (720 W, 35 s)	*L. acidophilus* PTCC 1643	Milk	Higher *L. acidophilus* count was observed in microwave and ultrasound–microwave-treated samples	Ultrasonicated microwaved samples exhibited higher antioxidant and anticancer activities	Gholamhosseinpour and Hashemi (2019)
HHP (600 MPa, 5 min)	*L. plantarum* LP299V	Mixed carrot and mango juice	Viability of probiotic strains maintained (35 days)	Maintained β-carotene content, antioxidant activity and sensory properties of the juices	Oliveira et al. (2020)
PEF (84, 126, 400, and 600 V. 100 μs)	*L. bulgaricus* CFL1	MRS broth	The application of high PEF intensities (above 285 V/cm) results in biphasic growth with the presence of two maximum specific growth rates	-	Peng et al. (2020)
PEF (0.3 kV/cm, 100 μs and 5 s, 21°C)	*L. plantarum* WCFS1, *L. plantarum* LMG23545, *L. diacetylactis* FM03 and *L. acidophilus* NCFM	Skim milk	PEF pretreatment increased survival after spray drying and storage	-	Vaessen et al. (2020)
High-pressure homogenization (HPH, 20 and 100 MPa)	*L. salivarus* CECT 4063	Mandarin juice	20 MPa HPH treatment maintained probiotic viability at ~6.8 log CFU/mL after 10 days of refrigerated storage	100 MPa treatment increased cell hydrophobicity	Betoret et al. (2017)

### 5.1 Ultrasound-assisted technologies

US employs sound waves with frequencies exceeding the range of human hearing (>18 kHz). These waves are divided into three primary categories based on their frequency: diagnostic (>1 MHz), high-frequency (20 kHz-2 MHz), and power (20–100 kHz). Cavitation is the basis of ultrasound. When sound waves and mechanical vibrations are applied to a liquid medium, alternating cycles of expansion and compression occur. During the expansion phase, high-intensity ultrasonic vibrations generate tiny bubbles that eventually combine. In the latter phase, the bubbles approach their threshold size and volume, resulting in a dramatic collapse over time due to their inability to absorb further energy. Cavitation describes the progressive cycle of bubble development, expansion, and implosion. During implosion, the bubbles release a large amount of energy, creating a surge in temperature (approximately 5,000 K) and pressure (estimated 50,000 kPa), resulting in cell breakage, particle dispersion, and localized sterilization (Munekata et al., 2020). Numerous studies have examined the lethal effects of sonication on microorganisms. Although high-intensity sonication can exert lethal effects, controlled or sub-lethal ultrasound treatments have been shown to stimulate microbial metabolism and fermentation performance. Several studies report accelerated fermentation kinetics and improved probiotic viability in dairy and plant-based systems following ultrasound-assisted treatments (Akdeniz and Akalın, 2023; Bolívar-Jacobo et al., 2023; Brito et al., 2024; Mizuta et al., 2023). Mechanistically, mild ultrasound exposure may enhance nutrient diffusion, increase membrane permeability, and activate stress response pathways that improve tolerance to subsequent environmental stressors (Shokri et al., 2021; Wang et al., 2021). These effects are strongly dependent on treatment intensity, duration, frequency, and strain-specific characteristics.

Therefore, while excessive sonication may damage cell structures, optimized ultrasound parameters can function as a metabolic stimulation strategy, contributing to improved probiotic stability and enhanced functional properties of fermented foods.

#### 5.1.1 Effects of ultrasound-assisted methods on probiotic viability

Ultrasound is a well-known food processing technique. Its use in probiotic foods has been reported to improve functionality and viability, and even change the prebiotic structure, making it more accessible to probiotics. The efficacy of a treatment in terms of microbial viability is influenced by specific process characteristics, including power level, exposure time, amplitude, targeted microorganisms, and temperature. Recent research demonstrates that ultrasound treatment positively affects the growth and proliferation of probiotics in fermented dairy foods (Shokri et al., 2021). Microstreaming is the main factor influencing the ultrasonic phenomena present in probiotic cells. It simultaneously facilitates the transfer of nutrients and gases, encouraging probiotic growth without causing physical harm. Munekata et al. (2020) investigated how ultrasonic therapy affected the fused matrix of thyme and rosemary. The researchers discovered that ultrasonic exposure suppressed infections while increasing the growth of probiotics, resulting in strong antioxidant and anti-inflammatory effects. Akdeniz et al. (2024) stated that ultrasound treatment enhanced the growth of *S. thermophilus*, *L. bulgaricus,* and *B. lactis* BB-12 in yogurt samples. Meena et al. (2024) demonstrated that low-intensity ultrasound treatment enhances the proliferation of probiotic (*L. rhamnosus* GG) and lactic acid production in millet-based probiotic beverages. Research shows that ultrasound can help enhance the viability of probiotics. The use of ultrasound in production is an environmentally friendly process, and it is hypothesized that this technology will positively influence consumer preference in line with the findings. Nevertheless, it is critically important to quantify and regulate ultrasound parameters to prevent excessive cell permeability and cell death.

#### 5.1.2 Effects of ultrasound-assisted methods on probiotic functionality

The enhancement of product functionality following ultrasound treatment is primarily attributed to the stimulation of probiotic metabolic activity and the modulation of enzyme release, influencing lipolysis and proteolysis. High-intensity ultrasound has been associated with increased *β*-galactosidase activity, accelerated lactose hydrolysis, and enhanced trans-galactosylation reactions in probiotic systems.

The increased production of bioactive peptides and oligosaccharides in ultrasound-treated fermented milk resulted in improved sensory qualities and higher nutritional value. In plant-based matrices, ultrasound has been applied to stimulate probiotic growth and enhance isoflavone bioconversion in soymilk. Liu et al. (2018) reported that short treatment times (2–3 min) increased β-glucosidase activity, promoting more efficient bioconversion processes.

In a study, researchers indicated that ultrasound enhanced hydrophobicity by 20% and the adherence of probiotic bacteria to CaCo-2 cells by a factor of 10 (Racioppo et al., 2017). Recently, Santos et al. (2024) reported that ultrasound pre-treatment better preserved the antioxidant potential of probiotic avocado samples. In another study, ultrasonic treatment of probiotic yogurt reduced the relative proportion of saturated fatty acids by up to 19% while increasing polyunsaturated fatty acid (PUFA) ratios by up to 40%. The authors also reported improved *in vitro* protein digestibility and increased total amino acid content before and after digestion (Akdeniz et al., 2024). Bevilacqua et al. (2024) indicated that ultrasound treatment could be used to improve some surface properties and, for *L. plantarum* c19, it was used to improve biofilm stability. Consequently, these studies demonstrate that ultrasound technology can be a valuable tool for developing probiotic foods with enhanced functionality.

#### 5.1.3 Effects of ultrasound assisted methods on food functionality

The application of ultrasound improved the water-holding capacity and viscosity of a fermented finger millet beverage (Meena et al., 2024). Ultrasound application trapped water within proteins, altering the water-holding capacity of the fermented beverage. Enhanced antimicrobial activity of probiotics, improved adhesion to the mucosa, and increased colonization in the GIT were observed. The ultrasound technique used to formulate double emulsion microcapsules containing LAB was analyzed (Pandey et al., 2021). The probiotic remained viable within the double emulsion microcapsule, exhibiting prospects for improved intestinal delivery of probiotic bacteria through food systems. Researchers reported that applying ultrasound to a Baru almond beverage enhanced acidification during storage, attributed to increased *L. casei* activity. Ultrasound application affected the phenolic composition of the beverage by enhancing both concentration and bioaccessibility during storage of the fermented product. Similarly, increased levels of monounsaturated fatty acids (MUFA) and PUFA were observed with improved fatty acid bioaccessibility. The volatile composition also improved, resulting in higher sensorial qualities. Homogenization-like effects were observed in probiotic yogurts due to ultrasound application. Overall, ultrasound application reduced particle size with increasing sonication power; in particular, the particle size of milk fat globules was reduced, increasing casein interactions. Increased bonding among casein micelles resulted in an increased water-holding capacity of yogurts. Additionally, faster acid development and a reduction in pH were observed, attributed to enhanced hydrolysis and the release of intracellular enzymes. Furthermore, enhanced viscosity improved gel strength due to strong associations between milk fat globules and proteins following high-intensity sonication. It was also reported that including the probiotic culture in samples pretreated with ultrasound helped improve the sensory quality of the yogurt by overcoming limitations associated with the development of free sulfhydryl groups affecting flavor (Akdeniz and Akalın, 2023).

Ultrasound pretreatment, followed by incorporation of *B. animalis* spp. *lactis* B94 and subsequent drying, increased the drying efficiency of avocado slices (Santos et al., 2024). The dehydrated (15% moisture) avocado slices retained viable probiotic populations exceeding 6 log CFU/g, exhibiting probiotic properties.

Ultrasound treatment at 120 W/L in milk samples increased the production of exopolysaccharide (kefiran) and ascorbic acid during the first hour of application (Potoroko et al., 2018). Sonication enhanced the activity of microorganisms, which further resulted in increased ascorbic acid production. Both ascorbic acid and kefiran production led to heightened antioxidant activity in the milk sample. In goat milk, ultrasound application for yogurt development helped improve major limitations such as firmness, consistency, and viscosity (Delgado et al., 2020). Ultrasound significantly reduced lactose content and increased glucose and galactose content with increased sonication time, an effect attributed to induced hydrolysis (Gholamhosseinpour and Hashemi, 2019). Yogurt produced from ultrasound-treated milk showed altered soluble protein profiles and increased amino acid content by enhancing protein hydrolysis, thereby improving the digestion of probiotic yogurts (Akdeniz et al., 2024).

#### 5.1.4 Effects of ultrasound on fermentation time

Meena et al. (2024) evaluated the influence of ultrasound application, before or after inoculation of *L. rhamnosus*, on the fermentation process for developing a finger millet-based probiotic beverage. Applying ultrasound (0.29 W/mm2 intensity for 2.63 min.) post-inoculation lowered fermentation time from 8.58 h to 2.52 h. Ultrasound significantly improved the fermentation of the finger millet-based probiotic beverage by reducing and disrupting particle size. Low-intensity ultrasound (23 kHz, 10 *μ* amplitude) increased the cell count of *L. brevis* (LMG 6906) by 1.09 log CFU/mL by enhancing fermentation and biomass production. Ultrasound accelerated the fermentation process by promoting growth rate, waste removal, and nutrient transport across the membrane (Shokri et al., 2021). A significant reduction in fermentation time (13.6%) and an increase in probiotic count was observed for the fermentation of a Baru almond beverage when ultrasound was applied before fermentation (dos Santos Rocha et al., 2023).

The positive influence of ultrasound application (640 J/mL) on fermentation time was observed in fermented milk (Brito et al., 2024). The 30-min reduction in time due to high-intensity ultrasound application enhanced permeability, leading to increased bacterial growth and improved fermentation. Treatment effects were also observed during the storage period, with LAB populations increasing after 28 days. The reduction of fermentation time for probiotic yogurts was attributed to increased activity of fermentative enzymes, improved culture viability, and increased acid production, resulting in faster fermentation (Akdeniz and Akalın, 2023). Sonication as a pretreatment for milk used in fermented milk production by *L. plantarum* AF1 resulted in the highest growth over a 24-h fermentation period, attributed to increased growth from the release of intracellular enzymes (Gholamhosseinpour and Hashemi, 2019). High-intensity ultrasound exhibited a positive interaction with the composition of oat fermentation compounds, increasing probiotic proliferation and indicating enhanced fermentation (Herrera-Ponce et al., 2022). High-intensity ultrasound (20 kHz) applied at 30% amplitude for 3 min resulted in maximum growth of *L. acidophilus* and *L. helveticus*, enhancing their fermentation activity (Bolívar-Jacobo et al., 2023). Specifically, an increase in β-galactosidase enzyme activity correlated with the fermentative capacity of the evaluated bacteria.

### 5.2 High hydrostatic pressure

HHP, also referred to as high-pressure processing, pascalization, or cold pasteurization, is a non-thermal preservation technique that inactivates harmful vegetative spoilage microorganisms by applying pressures typically ranging from 100 to 800 MPa (Vieira et al., 2024).

The preservation mechanism of HHP involves structural and functional alterations at the cellular level, including membrane permeabilization, protein denaturation, enzyme inactivation, and ribosome dissociation, which collectively impair microbial metabolic activity (Pankiewicz et al., 2020; Hao et al., 2021).

Three principles explain the preservation process: (i) the Le Chatelier principle, which states that the temperature range is 5–10 °C; that is, a pressure of 600 MPa cannot be higher than the temperature of water at 30 °C; (ii) the isostatic principle, which guides the application of consistent pressure across the food product, irrespective of its size or shape; and (iii) the microscopic ordering principle, which influences molecular structure and chemical reactions and increases with pressure at a constant temperature. Its main advantage over thermal processing is that it eliminates the need for high temperatures, thereby lowering the energy required for heating and cooling. Second, HHP is applied to food products in their final packaging, limiting the possibility of secondary contamination and re-use of the pressure medium. Conversely, recent years have seen investigations into the potential of HHP as a non-thermal technology to enhance the functional properties of products without compromising the biological activity of probiotics.

#### 5.2.1 Effects of high hydrostatic pressure on probiotic viability

The use of HHP in manufacturing probiotic foods is a subject of considerable research due to its promise as a non-thermal alternative for achieving pasteurization parameters without compromising probiotic viability (Vieira et al., 2024). Pressure treatments induce various changes in microbial cells, such as alterations in cell morphology and membrane, suppression of protein and enzyme synthesis, disruption of genetic mechanisms like transcription and translation, and changes in cellular processes vital for reproduction and survival. Conversely, some studies indicate that HHP successfully eliminates pathogens while maintaining probiotic viability. Tsevdou et al. (2020) investigated the effect of HHP (100–400 MPa at temperatures between 20 and 40 °C) on probiotic strains (*L. casei* and *B. bifidum*) added to yogurt. Findings suggested that a pressure range of 200–300 MPa improved strain survivability during a 28-day storage period. Nevertheless, existing literature has not fully addressed the relationship between HHP technology and its effects on probiotic foods. Accordingly, further research is required to determine the impact of this treatment on probiotic viability across various food matrices.

#### 5.2.2 Effects of high hydrostatic pressure on probiotic functionality

Applying low pressure directly to probiotic cells revealed strain-dependent effects on their capacity to enhance desired functional properties. Recently, Salas-Millán et al. (2024) found that HHP treatment (200 MPa, 10 min) of a lactofermented broccoli leaf beverage maintained the stability of its highest sulforaphane content (4.38–8.82 mg/L) and its antidiabetic potential during refrigeration.

These findings indicate that HHP enhances certain functional attributes of probiotics, underscoring its potential in the probiotic food industry. Nevertheless, more recent research is required to demonstrate the effects of this treatment on the functional properties of probiotics and probiotic foods.

#### 5.2.3 Effects of high hydrostatic pressure on food functionality

In plant-based yogurts, HHP application (600 MPa for 5 min) resulted in yogurts with viscosity and viscoelastic properties similar to dairy-based products (Sim et al., 2020). Considering the influence of different pressure applications in yogurt, 200 MPa pressure improved textural quality without hampering probiotic viability (Tsevdou et al., 2020). Applying high pressure (150 MPa) to homogenized protein encapsulation of probiotic cultures led to the development of a consistent, low-moisture probiotic product. The spray-dried cultures exhibited lower moisture content, particle size, and water activity due to changes in the protein matrix induced by pressure application (Mis-Solval et al., 2019).

Application of high pressure (600 MPa) lowered the psychotropic count to <1 log CFU/mL, while in the control sample, this value increased to 5.02 log CFU/mL after 35 days of storage (Oliveira et al., 2020). During storage, pH reduced and acidity was maintained in pressure-processed samples and increased in control samples, suggesting the influence of microbial growth. Similarly, retention of antioxidant capacity (α-carotene and β-carotene) and inhibition of enzymatic activity (polyphenol oxidase) were observed in samples treated with high pressure. Also, the sensory acceptability was higher for samples processed with high pressure. High-pressure (70 MPa) homogenization was evaluated for enhancing the survival and *in vitro* digestion of *L. salivarius* as a probiotic for fruit matrix (Ester et al., 2019). High-pressure homogenization resulted in effective encapsulation of the probiotic, improving its survival by 20% for 30 days of storage compared to non-encapsulated. Additionally, high-pressure application improved gastrointestinal survivability, enhancing target delivery and ensuring probiotic effect.

High pressure (600 MPa for 3 min) applied after fermentation and maturation in dry fermented meat sausage increased pathogen inactivation, lowered pH, and reduced water activity early on, resulting in quick product development without affecting its quality (Balamurugan et al., 2020). The impact of high-pressure processing on sliced ham, followed by coating with a probiotic alginate-based film, affected pH values and sensory quality. The growth of the probiotic favored the sensory quality of ham, making it more sensorially acceptable.

#### 5.2.4 Effects of high hydrostatic pressure on fermentation time

The application of high pressures at 20 and 100 MPa in mandarin juice with the addition of *L. salivarius* increased the proliferation of the probiotic (Betoret et al., 2017). High-pressure application resulted in small-sized cloud particles due to high-pressure homogenization (HPH). The surface properties of the microorganisms changed, which resulted in increased hydrophobicity. Compared to high pressure (100 MPa), the lower pressure (20 MPa) application maintained the probiotic requirement of the juice for 10 days. Considering the high capacity of high-pressure processing to inactivate microorganisms, it was suggested to include a higher concentration of probiotic during the formulation of plant-based yogurts by pressure application (Sim et al., 2020).

Considering the susceptibility of probiotics to high pressure (100–400 MPa), Tsevdou et al. (2020) evaluated the impact of different pressure treatments on the quality of probiotic yogurt produced with *B. bifidum* and *L. casei*. The starter culture exhibited a reduced count with increased pressure, with complete inactivation at 400 MPa. However, at a slightly lower pressure (300 MPa), probiotic survival was reduced but remained above the required level of 7 log CFU/g. Similarly, Yang et al. (2021) evaluated the impact of pressure acclimation on LAB strains (*P. acidilactici*, *L. brevis*, *L. plantarum*, and *L. curvatus*). The fermentation performance of *L. plantarum* and *L. curvatus* was enhanced, with survival ratios of 138 and 1,222, respectively. Pressure acclimation improved the acid-producing capacity of the LAB strains, suggesting better fermentation capacity (Yang et al., 2021). Considering the reduced viability of probiotics during spray drying, Mis-Solval et al. (2019) evaluated the impact of ultra-high-pressure homogenization of encapsulated proteins on the survivability of probiotics (*L. plantarum* NRRL B-1927). The encapsulation of probiotics resulted in greater survival.

Application of high pressure (600 MPa) did not negatively affect the viability of *L. plantarum* in either fresh or stored samples. On the contrary, counts increased from 8.2 to 9.0 log CFU/mL after 35 days of storage, thereby enhancing the probiotic population (Oliveira et al. 2020). Application of high pressure (600 MPa for 3 min) reduced the time required to produce dry fermented meat sausages (Balamurugan et al., 2020).

### 5.3 Pulsed electric field

The pulsed electric field (PEF) is a revolutionary approach for inactivating microorganisms using strong electric pulses. Using the PEF approach, liquid or semi-liquid foods are held between two electrodes for micro- to millisecond periods and subjected to brief, high-voltage pulses (typically 20–80 kV/cm). The mechanism of microbial inactivation by PEF involves electroporation, where electric pulses create pores in the microbial cell membrane, resulting in cell rupture and subsequent extrusion of cell contents as well as intrusion of surrounding media. Technical parameters like the number, duration, and intensity of pulses; microbial parameters like the growth curve phase and microbial characteristics (e.g., spore-forming ability); and product factors like pH, temperature, ionic force, electrical conductivity, and the presence of antimicrobial compounds are the main factors that determine how PEF affects microorganisms.

#### 5.3.1 Effects of pulsed electric field on probiotic viability

There is a lack of research on the use of PEF in probiotic foods. Sotelo et al. (2018) found that PEF treatment increased the growth of *L. acidophilus* ATCC 1643 in red cherries. Furthermore, Al Daccache et al. (2020) examined the fermentation of *Hanseniaspora* spp. isolated from Lebanese apples with PEF assistance (72–285 V/cm) and found that the maximum growth rate was achieved with an electric field strength of 285 V/cm applied in both the lag and log phases. PEF treatment reduced fermentation time while boosting biomass concentration. Spray-dried bacteria with an internal trehalose level of ≥3.5 μg/107 CFU had greater survival rates, up to 10.1 μg/107 CFU (above 100%), according to Gong et al. (2020). Electroporation at 2.5 kV/cm raised the survival rate from 38 to 61% following spray drying. Thus, electroporation of trehalose into LAB prior to spray drying can improve the vitality of microbial cells. Pankiewicz et al. (2020) found that PEF increased the survival of *L. rhamnosus* B442 in ice cream. Kanafusa et al. (2021) stated that nanosecond-scale PEF treatment increased metabolite production without affecting cell counts in fermented watermelon juice. Recently, Miranda-Mejía et al. (2024) investigated the effect of low-intensity PEF pre-treatment on yogurt starter cultures. They found that PEF could stimulate LAB metabolism and increase the lactic acid concentration (0.76–0.90%).

Collectively, these findings indicate that appropriately optimized PEF treatments may enhance probiotic growth, metabolite production, and survival in selected food systems while maintaining microbial viability. Nevertheless, further research is required to ascertain whether this technology facilitates more efficient and sustainable probiotic food production with high probiotic viability.

#### 5.3.2 Effects of pulsed electric field on probiotic functionality

PEF treatment has been explored as a sub-lethal strategy to modulate probiotic physiology and functionality. The observed improvements are attributed to transient membrane permeabilization and the activation of stress response pathways, which may enhance metabolic efficiency and adaptive capacity (Pankiewicz et al., 2020; Ma et al., 2023). Controlled electroporation can facilitate ion transport and nutrient uptake, potentially stimulating enzymatic activity and fermentation performance. Recent studies indicate that moderate PEF treatments may enhance probiotic robustness without compromising structural integrity when electrical field strength, pulse duration, and treatment time are carefully optimized (Brito et al., 2024; Vieira et al., 2024). However, excessive electric field intensity can lead to irreversible membrane disruption and viability loss. Therefore, while mild PEF pre-treatment shows potential as a functional modulation strategy, further investigation is required to standardize processing parameters and elucidate strain-specific responses before large-scale industrial application.

#### 5.3.3 Effects of pulsed electric field on food functionality

PEF technology, aside from affecting the viability of probiotics in food products, can also affect the nutritional and sensory properties of food while enhancing its functional qualities. The electroporation effect of cellular membranes modifies structural and biochemical attributes of food components without significant heating. Recent studies have demonstrated PEF’s efficacy in improving various aspects of food product functionality, such as texture, nutrient bioavailability, or antioxidant properties (Table 4).

**Table 4:** Examples of recent applications of PEF to improve food functionality.

Product	Effect	Reference
Beef meat	Decreased cutting strength, hardness, and chewiness Improved meat tenderization Slight increase in lipid oxidation and reduced lightness were observed after treatment	Jeong et al. (2023)
Chicken breast	PEF was combined with ultrasound treatment Reduced hardness, adhesiveness, and chewiness Improved meat tenderization Reduced cooking losses Higher release of flavor-related amino acids No effect on color	Roobab et al. (2024)
Beef meat	Reduced marination time Improved dispersity of NaCl and tenderness No effect on color and cooking loss	Zhang et al. (2022)
Potatoes	Reduced texture degradation during cooking PEF in combination with Ca^2+^ solution showed enhanced effects on texture stability PEF reduced potato pectin methylestrification PEF improved Ca^2+^ crosslinking	Moens et al. (2021)
Orange, pomelo, lemon	Increased juice yield by 25–59% Improved extraction of polyphenols using the solvent method	El Kantar et al. (2018)
Sugar beet	Increased cell disintegration Decrease in viscoelastic properties	Rezaee et al. (2019)
Olive leaves	Improved polyphenol extraction Extraction efficiency depended on solvent type	Pappas et al. (2021)
Potato peel	PEF prior to solid–liquid extraction improved extraction yield PEF allowed for reduced extraction time, temperature, and solvent use PEF extracts showed higher antioxidant power PEF did not cause any significant degradation of main bioactive polyphenols	Frontuto et al. (2019)
Green tea	PEF improved extraction yield PEF extracts had higher antioxidant power PEF assisted extraction caused significantly lower greenhouse gas emissions than maceration, ultrasound-assisted, or microwave-assisted extraction	Jara-Quijada et al. (2024)
Apple juice	Improved inactivation of polyphenol oxidase	Samaranayake et al. (2022)
Portobello mushroom	Decreased texture and color deterioration Inhibition of lipid oxidation and polyphenolic compound degradation Improved inactivation of polyphenol oxidase	Yan et al. (2020)
Soy protein isolate	PEF was combined with pH shifting Improved protein solubility, emulsifying properties, and foaming properties	Wang et al. (2023)

PEF has been thoroughly investigated for its potential to enhance food texture by disrupting cell walls and facilitating the release of intracellular components. PEF can improve meat tenderization, thereby enhancing its sensory and quality properties. The mechanism involves the electroporation of cell membranes, which disrupts sarcolemma and increases the permeability of intracellular structures. This improves the activity and release of natural proteases like calpains and cathepsins, leading to the degradation of myofibrillar proteins such as troponin-T or desmin, and thus improving meat tenderness (Jeong et al., 2023). PEF treatment also enhances the water-holding capacity of meat by causing the denaturation and partial solubilization of structural proteins, which reduces drip loss during cooking or storage (Roobab et al., 2024).

Moreover, the increased membrane permeability and wider gaps between muscle bundles improve the mass transfer of marinades and brine, allowing them to penetrate deeper and more uniformly into the meat. The disruption of cellular structures facilitates the diffusion of salts, sugars, and spices, significantly reducing marination time compared to conventional methods (Zhang et al., 2022).

Beyond meat, PEF can also improve the texture of plant-based foods, reducing texture deterioration during subsequent thermal treatment in vegetables such as carrots or potatoes (Moens et al., 2021). Moreover, membrane disruption enhances the extraction of various bioactive compounds. As shown by El Kantar et al. (2018), PEF improved juice yield and polyphenol extraction from lemon, pomelo, and oranges. Similar findings with improved polyphenol extraction have been observed for olive leaves (*Oleae folium*), potato peel (*Solani tuberosi cortex*), and green tea (*Camelliae sinensis*) (Frontuto et al., 2019; Jara-Quijada et al., 2024; Pappas et al., 2021). In addition to improving extraction yield, extracts obtained through PEF-assisted extraction show higher antioxidant power, which may be due to improved inactivation of enzymes such as polyphenol oxidase (Samaranayake et al., 2022; Yan et al., 2020). The inhibition of polyphenol oxidase by PEF improves the storage quality of many fruits and vegetables by inhibiting browning, as demonstrated for bananas, pineapple, and mushrooms (Moura et al., 2024).

PEF can also affect the functional properties of food matrices. By altering the secondary and tertiary structures of proteins, PEF induces changes in their solubility, emulsifying capacity, and foaming properties. These changes result from the unfolding and partial denaturation of proteins, which expose hydrophobic and functional groups, enhancing interactions with other food components (Taha et al., 2022). This was shown in a study by Wang et al. (2023), who observed that PEF combined with pH shifting improved the solubility of soy protein isolate from 26 to 70%. This, in turn, significantly improved the emulsifying and foaming properties of the protein isolate.

#### 5.3.4 Effects of pulsed electric field on fermentation time

Recent studies have shown that PEF can be utilized during the fermentation process. The use of PEF during this process can provide benefits such as reduced fermentation time and inactivation of spoilage microorganisms (Feng et al., 2022). Although PEF is known for its use against microorganisms, it can also be applied to improve fermentation kinetics if applied in sub-lethal doses, leading to a growth-stimulating effect (Pankiewicz et al., 2020; Ma et al., 2023). The distinction between lethal and sub-lethal doses depends on the power of the electric field, treatment time, pulse frequency, or even geometry and polarity of the pulses (Demir et al., 2023).

PEF has been particularly effective in reducing fermentation time in dairy and non-dairy products. In dairy fermentations, such as yogurt production, PEF treatment of milk prior to inoculation with starter cultures has been shown to reduce fermentation time while maintaining desired product quality attributes (Miranda-Mejía et al., 2024). For instance, applying PEF to milk can lead to improved growth of LAB and a faster decrease in milk pH. This improves product safety and the aggregation of casein, which in turn leads to the formation of a gel network (Chanos et al., 2020). This effect is attributed to the reversible electroporation effect that occurs due to PEF at sub-lethal doses, resulting in temporarily increased permeability of microbial cell membranes induced by PEF, which facilitates nutrient uptake and metabolic activity (Demir et al., 2023).

In alcoholic fermentations, PEF has been used to stimulate yeast activity in wine, beer or cider production (Al Daccache et al., 2020). Studies have shown that PEF at lethal doses can also be used to inactivate wild yeasts in grape must and therefore improve the fermentation kinetics by starter culture. As shown by Ricci et al. (2020), PEF treatment of grape must has a significant impact on the quality of the produced wine, improving color intensity and polyphenolic content.

### 5.4 Other innovative techniques

**Figure 4:**
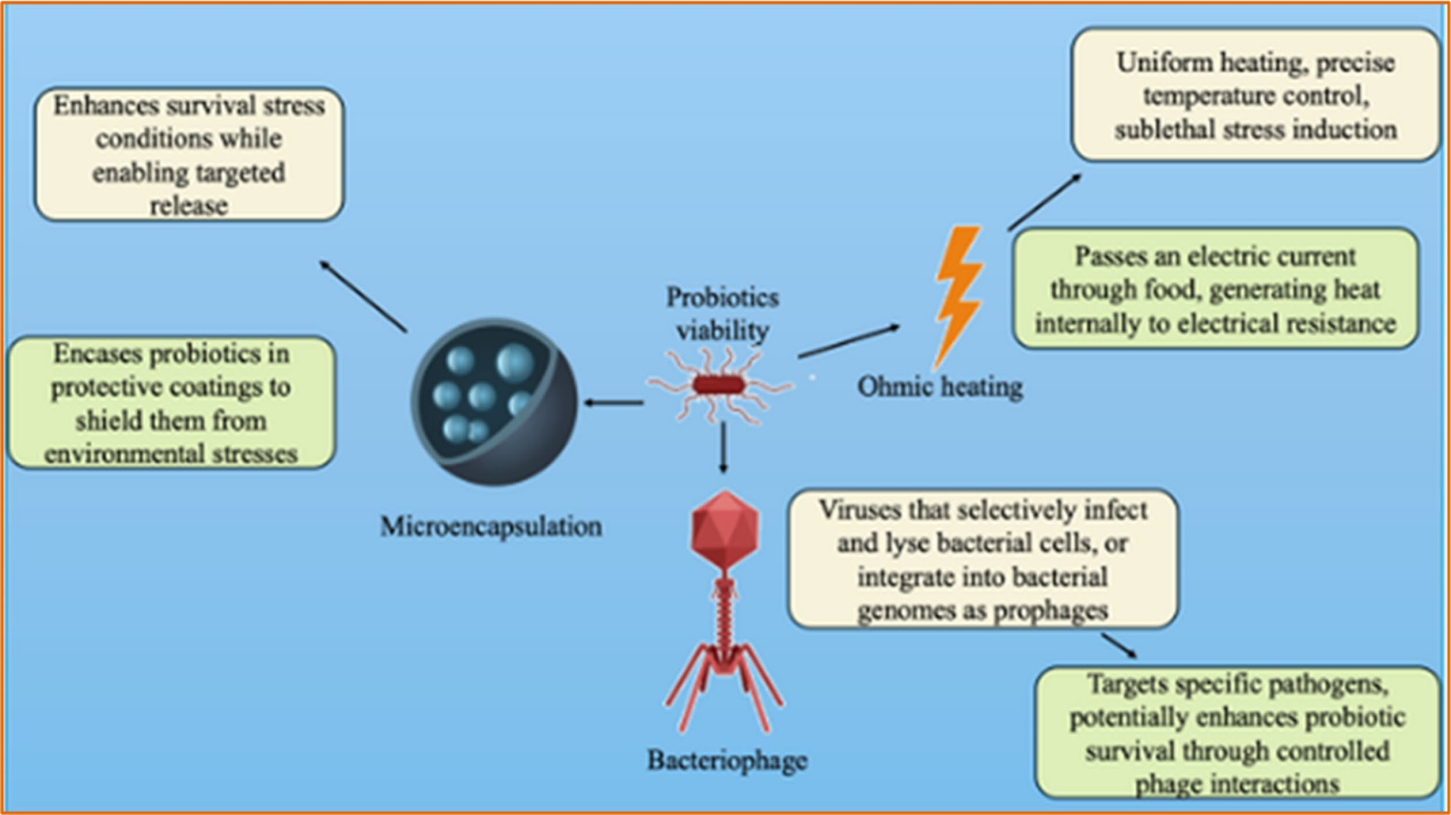
Other non-thermal methods for enhancing probiotics viability (created with BioRender.com).

In addition to the non-thermal technologies discussed above, several complementary strategies have been developed to enhance probiotic stability and functionality in food systems (Figure 4).

#### 5.4.1 Synbiotics

Probiotics are defined as live microorganisms that confer health benefits when administered in adequate amounts (Hill et al., 2014). However, their technological application is often limited by instability during processing, storage, and gastrointestinal transit.

As a complementary approach, prebiotics such as inulin, fructooligosaccharides (FOS), galactooligosaccharides (GOS), and xylooligosaccharides (XOS) have been extensively incorporated into probiotic food systems due to their ability to selectively stimulate beneficial microbial populations and support microbiome modulation. Unlike general dietary fibers, these compounds are preferentially utilized by specific microbial groups, thereby contributing to improved probiotic functionality and gut microbial balance.

The combination of probiotics and prebiotics, known as synbiotics, was first described by Gibson and Roberfroid (1995) and subsequently refined by the International Scientific Association for Probiotics and Prebiotics (ISAPP; Swanson et al., 2020). Synbiotics may exert synergistic effects by simultaneously delivering viable microorganisms and their preferential substrates, enhancing survival, colonization potential, and metabolic performance.

From a mechanistic perspective, probiotic utilization of prebiotics depends on substrate structure and strain-specific carbohydrate metabolism pathways. Short-chain FOS are typically fermented rapidly in the proximal colon, whereas longer-chain inulin persists in distal regions, influencing fermentation dynamics and metabolite production (Zhang et al., 2022).

Beyond physiological benefits, such as modulation of bile salt metabolism, immune regulation, and anti-inflammatory activity, synbiotics also provide technological advantages. Prebiotic matrices can act as protective carriers, improving probiotic stability during storage and gastrointestinal exposure.

Encapsulation techniques, for example, have emerged as a promising tool for the effective delivery of synbiotics. Malos et al. (2025) demonstrated that microcapsules containing *L. plantarum* with FOS coated in chitosan and alginate improved survival to log 8 CFU/mL under simulated gastrointestinal conditions. Similarly, Thinkohkaew et al. (2025) reported that 4% FOS in chitosan-coated alginate/gellan gum microcapsules achieved 83.36% survival after freeze-drying and retained ~6.4 log CFU/g after digestion, sustaining viability for 90 days at 4 °C. These findings highlight the robustness of encapsulation as an approach to overcoming key technological barriers. In summary, synbiotics represent a synergistic strategy that enhances probiotic viability, selectively stimulates beneficial microbiota, and adds value to functional food design. However, challenges remain, such as strain-specific responses, dosage optimization, and regulatory uncertainties. Nevertheless, the integration of synbiotics, particularly through advanced encapsulation methods, shows great potential for scalable applications in food and therapeutic contexts.

#### 5.4.2 Microencapsulation

Microencapsulation involves entrapping probiotic cells within protective matrices to shield them from adverse environmental conditions during processing, storage, and gastrointestinal transit. By forming microcapsules around viable cells, this technique enhances probiotic stability across diverse food matrices.

The protective mechanism of microencapsulation is achieved through improved thermotolerance of probiotics, enabling them to withstand higher temperatures that would otherwise reduce their viability. For instance, microencapsulation has been shown to increase the survival rates of probiotics during thermal processing, such as spray-drying or mild heat treatment (Bagdat et al., 2024). It should be mentioned, though, that even microencapsulated bacteria will not survive at higher temperatures for prolonged periods (Hao et al., 2021; Mahmoud et al., 2020). Microencapsulation can also safeguard probiotics from stomach acid or intestinal bile salts, improving the chances of their successful delivery to the gut. Moreover, microencapsulation can improve the stability of probiotics during storage by protecting them from oxygen, moisture, and other environmental factors that may compromise their viability over time (Rajam and Subramanian, 2022).

Several encapsulation techniques are commonly applied. Emulsion-based methods entrap probiotics within dispersed droplets that are subsequently solidified, providing a relatively simple and scalable approach. Spray-drying encapsulation involves atomization of probiotic suspensions with carrier materials such as maltodextrin or whey proteins, producing stable powdered formulations suitable for dry food systems (Barajas-Álvarez et al., 2022). Freeze-drying (lyophilization) combined with protective agents remains widely used for thermosensitive strains, as it preserves structural integrity during dehydration (Mahmud et al., 2022).

Microencapsulation can also be integrated with other non-thermal methods to further improve probiotic viability and technological efficiency. One common combination involves using non-thermal methods, such as HPP, to eliminate the food product’s native microflora before adding microencapsulated probiotic strains. This reduces competitiveness and improves product shelf life and stability (Worametrachanon et al., 2014). However, adding probiotics after HPP treatment can be problematic because HPP is primarily a post-packaging preservation technique, and packages are generally not reopened after processing. To address this limitation, McGillin et al. (2022) developed a proof-of-concept system where cocoa butter microencapsulated freeze-dried probiotic strains were incorporated into the product prior to HPP treatment. After processing, controlled heating of the sealed packaged product to 40 °C allowed the cocoa butter matrix to melt, enabling rehydration and subsequent proliferation of the encapsulated bacterial cells without reopening the package. This method yielded 8–9 CFU/g of viable probiotic cells while reducing the native microbiota and extending shelf life.

Other non-thermal methods can enhance microcapsule properties, thereby affecting probiotic survivability. Sun et al. (2024) applied cold plasma to modify the surface charge and structure of alginate nano-montmorillonite, which resulted in a denser structure and improved viability of encapsulated *L. kefiranofaciens* after exposure to simulated digestion.

However, not all studies show a beneficial effect of method combinations. Giordano et al. (2022) reported that combining sonication with microencapsulation did not further improve the viability or attenuation effect (negative changes in pH and sensory scores) of stored tomato juice.

Therefore, microencapsulation can be a powerful tool for improving probiotic viability in regular food products and enhancing survivability during other processing and preservation steps.

#### 5.4.3 Ohmic heating

Ohmic heating involves passing an electric current through food, causing rapid and uniform heating due to the food’s electrical resistance. While this method can inactivate pathogens and spoilage organisms, it can also damage probiotic bacteria. However, as reviewed by Pereira et al. (2018), ohmic heating can also be applied in sub-lethal conditions to enhance bacterial growth, bacteriocin production, and improve the fermentation process. One advantage of ohmic heating is the precise control over treatment parameters, which can affect probiotic viability during sub-lethal conditioning. In addition to improving fermentation, sub-lethal ohmic heating can be used for probiotic preconditioning or sub-lethal stress application. This process exposes probiotics to mild stresses to induce a cellular stress response, which in turn promotes the production of cell components such as heat-shock proteins. It has been shown that sub-lethal exposure to mild temperature shock of *L. casei* strains promotes their survival during later heat-drying steps (Bommasamudram et al., 2022). Therefore, sub-lethal ohmic heating can enhance stress tolerance by inducing the synthesis of protective proteins and promoting structural adaptations in the cell membrane, allowing probiotics to maintain their viability and efficacy when incorporated into functional foods (Pereira et al., 2018). Moreover, sub-lethal ohmic heating is being explored for its potential in producing paraprobiotics—non-viable probiotics with retained health-promoting properties. Ohmic heating allows for careful modulation of heating parameters to inactivate cells without destroying bioactive components (Siciliano et al., 2021).

#### 5.4.4 Bacteriophage application

Bacteriophages (phages) are viruses that specifically infect bacteria and can influence the stability and viability of probiotic cultures in food systems and the gastrointestinal tract (Rogovski et al., 2021). Their presence may affect probiotic performance either negatively, through phage-induced lysis of beneficial strains, or positively, through selective modulation of competing or pathogenic bacterial populations.

Phages can impact probiotic populations through mechanisms such as lysogeny, where the phage integrates its genetic material into the host bacterium’s genome, known as a prophage. This can lead to disadvantages or advantages, depending on the nature of the prophage. However, researchers are exploring the potential to engineer prophages using carefully selected phages, potentially conferring advantages such as enhanced stress resistance or metabolic capabilities. This interaction can enhance the survival and functionality of probiotics within the GIT (Qiao et al., 2020). However, the presence of lytic phages, which actively replicate and cause the destruction of bacterial cells, poses a challenge to maintaining probiotic viability in both food products and the gut environment. Phage-induced lysis can lead to significant reductions in probiotic populations, undermining their health benefits. To mitigate this, strategies such as the development of phage-resistant probiotic strains or the application of protective encapsulation techniques are being explored. Encapsulation can shield probiotics from phage attacks, thereby preserving their viability during storage and after ingestion (Wen et al., 2024).

Overall, bacteriophages represent both a potential risk factor for probiotic viability and a promising microbiological management tool in probiotic food systems. Understanding phage-probiotic interactions may contribute to the development of more stable fermentation processes and improved strategies for maintaining probiotic viability during processing and storage.

#### 5.4.5 Atmospheric cold plasma method

The atmospheric cold plasma (CP) method is a short-term food processing treatment conducted at low temperatures in various gas compositions with different parameters, such as frequency, treatment time, voltage, and electrode gap distance. In the food industry, CP can be utilized for raw material pretreatment to achieve advanced attributes; for example, it can inactivate pathogenic microorganisms on fermentation substrates without significant thermal degradation of sensitive nutrients. Furthermore, CP-induced mild oxidative stress can enhance the metabolic activity of starter cultures, resulting in improved fermentation performance (Souza et al., 2025).

The mild oxidative stress induced by CP can increase total phenol content and, consequently, antioxidant activity (Amiri et al., 2025). Additionally, CP treatment can induce structural changes in fiber, leading to increased SCFA content achieved by fermentation, as well as enhanced prebiotic activity and probiotic growth (Mehta et al., 2022). Similarly, Pina-Pérez et al. (2024) reported that CP degraded complex polysaccharides in *Arthrospira platensis* (Spirulina) into more fermentable substrates, increasing the prebiotic activity of *L. rhamnosus* and *Limosilactobacillus rhamnosus*. Sun et al. (2024) applied CP to modify the surface charge and structure of alginate nano-montmorillonite, which in turn resulted in a dense structure and improved viability of encapsulated *L. kefiranofaciens* after exposure to simulated digestion. CP also degraded toxic compounds (formic acid, acetic acid, furfural, etc.) in sulfuric acid-hydrolyzed sugarcane bagasse, leading to increased bioethanol production (from 0.25 to 0.65 g/L/h) in *Kluyveromyces marxianus* (Lin et al., 2020). In *Klebsiella pneumoniae*, dielectric barrier discharge plasma improved tolerance to glycerol and 1,3-propanediol production by inducing the activities of key enzymes: glycerol dehydratase, glycerol dehydrogenase, and 1,3-propanediol oxidoreductase (Dong et al., 2010). The same method also increased ethanol production in *Saccharomyces cerevisiae* (Dong et al., 2014).

Using atmospheric pressure glow discharge plasma, mutations were generated in *Streptomyces avermitilis* to increase antiparasitic avermectin B1a production (Wang et al., 2010).

### 5.5 Advantages of non-thermal technologies over thermal technologies

Non-thermal technologies, such as HHP, PEF, and US, offer several advantages over traditional thermal methods for processing food products, particularly those containing probiotics (Table 5). Thermal treatments can damage probiotics by disrupting cellular membranes, denaturing proteins, and reducing metabolic functionality, ultimately compromising their viability. Non-thermal methods, conversely, use mechanical, electrical, or pressure-based processes that inactivate pathogens and spoilage organisms while preserving probiotic viability and functionality. These technologies also retain the nutritional and sensory attributes of food, ensuring product quality and enhancing probiotic survival during processing and storage. By selectively inactivating harmful microorganisms without affecting probiotics, non-thermal technologies extend product shelf life while maintaining probiotic efficacy, a challenge difficult to achieve with thermal methods (Sotelo et al., 2018).

**Table 5:** Key benefits of non-thermal technologies as compared to thermal technologies.

Advantage	Non-thermal technologies	Thermal technologies
Probiotic viability	Preserves probiotic viability by avoiding heat-induced damage	Probiotics may be damaged by high temperatures, reducing survival
Nutritional quality	Retains nutritional content (vitamins, minerals, bioactive compounds)	Heat can degrade sensitive nutrients (e.g., vitamins)
Sensory properties	Maintains natural flavors, textures, and colors	Can alter flavor, texture, and color due to heat exposure
Selective microbial inactivation	Inactivates harmful microorganisms while preserving probiotics	May inactivate both harmful and beneficial microorganisms
Fermentation time	Reduces fermentation time (e.g., PEF, ultrasound)	Longer fermentation time due to slower microbial growth
Energy efficiency	Often more energy-efficient (e.g., PEF, HPP)	Energy-intensive, especially at high temperatures
Environmental impact	More sustainable, with less energy consumption	Higher energy consumption and associated carbon footprint
Product shelf life	Extends shelf life by preserving probiotics and food quality	Shortened shelf life due to loss of probiotics and altered food properties
Heat-sensitive products	Ideal for heat-sensitive products (e.g., dairy, juices)	Not suitable for heat-sensitive products as it causes damage
Consumer demand	Aligns with consumer preference for minimally processed foods	Perceived as heavily processed by consumers

Research has shown the potential of non-thermal technologies to enhance the quality and viability of probiotics in various food products. For instance, PEF has been found to improve the shelf life of probiotics in red cherries and ice cream while also reducing fermentation time in different food matrices (Al Daccache et al., 2020; Pankiewicz et al., 2020). PEF applied at sub-lethal doses has been shown to improve microbial growth and metabolism by temporarily increasing cell membrane permeability, which enhances nutrient uptake and accelerates fermentation (Taha et al., 2022). In dairy fermentations, PEF boosts LAB growth, accelerates pH reduction, and enhances casein aggregation, improving the texture and safety of yogurt (Miranda-Mejía et al., 2024; Morais et al., 2023). Similarly, in alcoholic fermentation, PEF enhances yeast activity, improving fermentation kinetics and wine quality (Al Daccache et al., 2020). High-pressure treatments, ranging from 20 to 100 MPa, have been demonstrated to increase probiotic proliferation, particularly in mandarin juice, where pressures around 20 MPa preserved probiotic viability for up to 10 days (Tsevdou et al., 2020). While high-pressure treatment at higher pressures (100–400 MPa) may inactivate probiotics, pressures around 300 MPa maintain survival above required levels, and pressure acclimation improves fermentation in LAB strains (Yang et al., 2021).

Ultrasound treatment, another non-thermal method, offers several benefits for probiotic foods by stimulating enzyme release and improving lipolysis, proteolysis, and fermentation. Ultrasound accelerates the secretion of β-galactosidase and enhances the production of bioactive peptides, improving both the nutritional and sensory properties of fermented products (Racioppo et al., 2023). Studies have shown that ultrasound can increase probiotic counts, reduce fermentation times, and improve the protein and fatty acid content of yogurts and beverages (Meena et al., 2024). Ultrasound is a useful method for enhancing the functionality of foods high in probiotics because it also increases the bacterial adhesion, hydrophobicity, and antioxidant potential of probiotics. The application of non-thermal methods, such as ultrasound and HPP, also offers flexibility for processing heat-sensitive products, including beverages, yogurts, and other functional foods, where traditional heat treatments often result in probiotic losses. Moreover, non-thermal technologies align with consumer demand for minimally processed foods, as they preserve natural flavors, textures, and nutrient profiles, offering a more sustainable and energy-efficient alternative to thermal methods. These advantages make non-thermal technologies a promising option for the development of probiotic-rich functional foods that maintain both microbial stability and high product quality throughout their shelf life.

### 5.6 Challenges for viability and functionality of probiotics

Despite recent developments, enhancing probiotic viability and functionality in food systems remains challenging across processing, storage, and gastrointestinal transit stages.

The first challenge relates to processing sensitivity. Many probiotic strains are susceptible to environmental stresses such as heat, oxygen exposure, mechanical forces, and pressure fluctuations. Although non-thermal technologies provide promising alternatives to conventional heat treatments, their effectiveness varies. For example, bacterial survival rates generally differ, and the effects of PEF on *L. rhamnosus* physicochemical parameters (content of dry matter, protein, fat, and carbohydrates, and melting rates) in ice creams (Pankiewicz et al., 2020), HHP on *L. plantarum* regarding total phenol content and antioxidant activity in fermented fruit juices (Ma et al., 2023), and US on *L. acidophilus* fermentation time of milk in yogurt (Brito et al., 2024) are all strain-dependent. This requires precise optimization of processing parameters to avoid sub-lethal damage or unintended inactivation.

The second challenge concerns storage stability. Probiotic viability may decline during shelf life due to oxygen exposure, moisture fluctuations, temperature variation, and post-acidification in fermented products (Wendel, 2022; Meybodi et al., 2020). Microencapsulation has been proposed as an effective protective strategy; however, it increases formulation complexity and production costs and may influence texture or sensory perception depending on the encapsulating material and food matrix compatibility (Yao et al. 2020; Camelo-Silva et al. 2022).

A third major challenge is survival during gastrointestinal transit. Probiotics must withstand acidic gastric conditions, bile salts, digestive enzymes, and osmotic stress to reach the colon in adequate numbers (Hill et al., 2014; Wendel, 2022). While strategies such as pre-conditioning and encapsulation may enhance survival, their protective efficacy remains strain-specific and influenced by host-related factors.

Regulatory and consumer-related aspects further complicate probiotic product development. Regulatory frameworks differ between regions and often require strain-specific clinical evidence before allowing health claims (Hill et al., 2014; Mukherjee et al. 2022; ISAPP, 2022). The lack of global harmonization in definitions, admissible claims, and safety evaluation criteria continues to create classification and labeling challenges.

Finally, industrial scale-up of innovative non-thermal technologies remains a technical and economic barrier. Technologies such as PEF and high-pressure processing require significant capital investment and specialized operational expertise, limiting widespread industrial adoption (Müller et al., 2020; Vieira et al., 2024).

Addressing these limitations requires multidisciplinary collaboration among food technologists, microbiologists, regulatory authorities, and industry stakeholders. Advances in strain selection, adaptive stress conditioning, encapsulation systems, and process optimization, combined with harmonized regulatory frameworks, will be critical to improving the long-term viability and functional reliability of probiotic foods.

### 5.7 Economic feasibility and scalability

Non-thermal techniques are recognized for their ability to produce high-quality products. However, they require significant capital investment. On the positive side, these techniques are environmentally friendly and cost-effective over the long term due to their prolonged operational periods. Additionally, non-thermal techniques typically require lower energy inputs as they do not involve heating. Despite their advantages, non-thermal processing can increase product costs, making such products suitable for premium customers. However, advancements in technology could help reduce these costs. In contrast, traditional methods often expose samples to thermal shock, which can diminish effectiveness, compromise quality, and lower stability. Furthermore, non-thermal methods reduce power consumption and the need for water and solvents, thereby lowering operational costs and enhancing energy efficiency. When considering industrial barriers, the commercial adoption of non-thermal processing methods faces several challenges. These include scaling up to large-volume production and improving processing capacity. The complexity of the equipment necessitates specialized training for operators. Nevertheless, with ongoing advancements in technology, equipment can be modified to better meet processing limitations and requirements.

### 5.8 Paraprobiotics and postbiotics: applications, stability, and health benefits

Recent evidence indicates that probiotics and their metabolites, whether live or non-viable, can exert health-promoting effects. As viability is not always necessary to trigger biological responses, the concept of paraprobiotics has been proposed as a broader category than traditional probiotics.

Paraprobiotics, according to Taverniti and Guglielmetti (2011), are non-viable bacterial cells or cell fragments that, when given in sufficient quantities, have positive health effects. Often termed “inactivated” or “ghost probiotics,” they are considered safer and more stable alternatives for use in food products. Their non-viable status provides advantages in terms of safety, storage stability, and reduced risk of microbial overgrowth in vulnerable populations. However, health effects remain strain-specific and must be clinically validated.

In contrast, postbiotics are bioactive metabolites or by-products of probiotics, including organic acids, SCFAs, exopolysaccharides, bacteriocins, and cell wall components (Aggarwal et al., 2022). These compounds are naturally present in fermented foods or can be intentionally incorporated into food matrices and packaging systems. Postbiotics have been associated with various biological activities, including immunomodulatory, anti-inflammatory, antioxidant, antimicrobial, antihypertensive, hypocholesterolemic, and anti-obesity effects, primarily attributed to their metabolites. The functional properties of postbiotics confer greater stability, texture, and palatability compared to probiotics, positively influencing the physicochemical and sensory characteristics of the final product and allowing their use as functional excipients. Moreover, postbiotics do not adversely alter sensory attributes; for instance, yogurt enriched with postbiotics was shown to avoid excessive acidification after fermentation.

Despite growing evidence of their benefits, there are currently no standardized regulations governing the use of paraprobiotics and postbiotics. Like probiotics, the health effects of paraprobiotics are strain-specific and must be validated in well-designed clinical trials. Nevertheless, both paraprobiotics and postbiotics exhibit biological activities, such as immunomodulatory, antioxidant, and antimicrobial effects, which are similar or complementary to those of live probiotics.

Several randomized controlled trials have confirmed the immunomodulatory potential of heat-inactivated *L. plantarum*. Lee et al. (2017) reported enhanced natural killer (NK) cell activity and increased IL-12, IgG1, and IF-*γ* concentrations after 12 weeks of yogurt consumption containing heat-treated strains. Similarly, Bae et al. (2022) and Hong et al. (2024) demonstrated that supplementation with paraprobiotic capsules improved innate immune responses, including NK cell activity and cytokine production, without adverse effects. These findings highlight paraprobiotics as safe and effective alternatives for strengthening host immunity.

Paraprobiotics have also been successfully incorporated into various food matrices without compromising product quality. For instance, the addition of *L. gasseri* to grape juice did not alter pH or acidity (Gholian et al., 2023), while inactivated *L. plantarum* in soy-based beverages enhanced antioxidant activity and phenolic content (Zahidah et al., 2024). Notably, strain selection remains critical: Parvarei et al. (2021) observed that dead cells of *B. lactis* BB-12 improved yogurt sensory attributes, whereas inactivated *L. acidophilus* reduced acceptability. Such results indicate that paraprobiotics not only maintain but may even improve sensory and functional qualities, supporting their integration into commercial functional foods.

From a technological standpoint, paraprobiotics and postbiotics offer clear advantages over live probiotics, as they exhibit greater stability during processing, storage, and passage through the GIT. Their robustness facilitates incorporation into diverse food products, functional ingredients, and therapeutic formulations. However, challenges remain regarding standardized definitions, mechanistic understanding, and regulatory frameworks, highlighting the need for further research and international harmonization.

## 6 Future perspectives

Future technologies aimed at maintaining probiotic viability in food products must adopt a multidisciplinary and adaptive approach to address challenges across processing, storage, and consumption stages. Innovations in strain selection will play a pivotal role, focusing on identifying and engineering probiotic strains with inherent resistance to environmental stresses such as heat, oxygen, and gastric acidity. Advanced genomic and proteomic tools can be utilized to pinpoint genetic markers for stress tolerance, enabling the development of robust strains tailored to specific food matrices. Non-thermal processing methods, such as HHP and PEF, offer promising alternatives to traditional thermal techniques. However, scaling up these technologies will require significant investment in cost-effective equipment and process optimization protocols to balance efficacy and economic feasibility. Furthermore, microencapsulation techniques must evolve to incorporate novel materials compatible with diverse food systems while maintaining sensory qualities and ensuring cost-effectiveness. The combination and optimization of HHP, PEF, US, CP, and ohmic heating techniques could be investigated in the future to increase the effectiveness of fermentations utilizing different strains. However, the unlikely but theoretically potential toxicity of residual plasma-generated compounds from CP techniques requires testing to guarantee safety.

Research should focus on expanding existing methods, optimizing microcapsule design, and addressing technological challenges to enable the development of novel functional foods. Synbiotics, combining probiotics with prebiotics, offer a promising avenue for enhancing gut microbiota modulation and health benefits. Identifying effective prebiotics to support probiotic survival in the GIT and utilizing sustainable resources for prebiotic extraction will be critical for future advancements. To ensure successful adoption, collaboration between academia, industry, and stakeholders is essential for fostering consumer awareness and driving innovation in functional foods.

## 7 Conclusion

Probiotic viability in food products is influenced by multiple interrelated factors, such as processing methods, food matrix composition, storage conditions, and environmental stressors. Addressing these factors is essential for creating probiotic foods that consistently deliver health benefits at the time of consumption. While thermal treatments are effective for pathogen inactivation, they often compromise probiotic survival due to membrane disruption and protein denaturation.

In contrast, non-thermal technologies such as HHP, PEF, US, CP, and ohmic heating preserve probiotic viability, maintain nutritional content, and minimize degradation of bioactive compounds. The composition of the food matrix significantly impacts probiotic stability, with factors such as pH, fat content, and the inclusion of prebiotics or encapsulation materials offering protective benefits. Maintaining probiotic levels across a product’s shelf life also requires optimizing storage conditions, such as temperature, humidity, and packaging.

Although numerous studies have investigated individual non-thermal technologies, the literature remains fragmented, with most reports focusing on single processing methods, specific strains, or isolated food matrices. Moreover, probiotic viability is often evaluated solely through culturability, without integrating functional or metabolic indicators, leading to inconsistencies in interpreting technological effectiveness. This lack of integrated evaluation represents a critical gap in current research. This review bridges this gap by comparatively synthesizing recent experimental evidence on non-thermal technologies, including HHP, PEF, ultrasound, and ohmic heating. It examines not only survival outcomes but also functional performance, metabolic enhancement, and matrix-dependent responses. By structuring the evidence across processing technologies, food systems, biological responses, and regulatory considerations, this review provides a multidimensional framework for understanding probiotic stability beyond simple CFU preservation. Furthermore, the integration of emerging concepts such as paraprobiotics and postbiotics within non-thermal processing contexts remains limited in the literature. This review highlights the potential of combining technological and biological strategies to enhance both stability and functionality, offering a more comprehensive approach to probiotic product development.

From a practical standpoint, these insights can assist the food industry in selecting strain-specific and matrix-appropriate mild processing technologies for the development of stable, high-quality probiotic products aligned with sustainability goals and consumer expectations. Nevertheless, several challenges remain, including strain-dependent technological responses, feasibility of industrial-scale implementation, regulatory harmonization, and standardization of viability assessment methodologies. Future research should therefore prioritize: (i) harmonization of viability and functionality assessment tools, (ii) development of strain-specific processing parameters, (iii) integration of encapsulation and non-thermal strategies, (iv) techno-economic and scalability evaluations, and (v) clearer regulatory pathways for probiotic, paraprobiotic, and postbiotic applications.

By addressing these critical gaps, the food industry can advance toward the production of probiotic products that consistently retain their health-promoting properties while meeting safety, quality, sustainability, and regulatory standards.

## References

[ref1] Afzaal M. Khan A. U. Saeed F. Ahmed A. Ahmad M. H. Maan A. A. . (2019). Functional exploration of free and encapsulated probiotic bacteria in yogurt and simulated gastrointestinal conditions. Food Sci. Nutr. 7, 3931–3940. doi: 10.1002/fsn3.1254, 31890171 PMC6924303

[ref9001] Aggarwal S. Sabharwal V. Kaushik P. Joshi A. Aayushi A. Suri M. (2022). Postbiotics: From emerging concept to application. Frontiers in Sustainable Food Systems, 6, 887642.

[ref2] Akdeniz V. Akalın A. S. (2023). Power ultrasound affects physicochemical, rheological and sensory characteristics of probiotic yoghurts. Int. Dairy J. 137:105530. doi: 10.1016/j.idairyj.2022.105530

[ref3] Akdeniz V. Öcal G. K. Armağan G. Akalın A. S. (2024). High-energy ultrasound improves culture activity, polyunsaturated fatty acids and in vitro protein digestibility in probiotic yogurt. Innov. Food Sci. Emerg. Technol. 92:103573. doi: 10.1016/j.ifset.2024.103573

[ref4] Al Daccache M. Koubaa M. Maroun R. G. Salameh D. Louka N. Vorobiev E. (2020). Pulsed electric field-assisted fermentation of *Hanseniaspora* sp. yeast isolated from Lebanese apples. Food Res. Int. 129:108840. doi: 10.1016/j.foodres.2019.108840, 32036887

[ref5] Alan Y. Keskin A. O. Sönmez M. (2025). Probiotic and functional characterization of newly isolated *Lactiplantibacillus plantarum* strains from human breast milk and proliferative inhibition potential of metabolites. Enzym. Microb. Technol. 182:110545. doi: 10.1016/j.enzmictec.2024.110545, 39546820

[ref6] Alinovi M. Bancalari E. Monica S. Del Vecchio L. Cirlini M. Chiavaro E. . (2025). Tailoring the physico-chemical properties and VOCs of pea-based fermented beverages through *Lactobacillus delbrueckii* subsp. *bulgaricus* and *Streptococcus thermophilus* fermentation. Food Res. Int. 209:116250. doi: 10.1016/j.foodres.2025.116250, 40253184

[ref7] Amiri M. Arab M. Mortazavian A. M. (2025). The effect of cold plasma treatment on the phenolic and flavonoid content and antioxidant activity of whole buckwheat grain and flour. Sci. Rep. 15:25132. doi: 10.1038/s41598-025-10281-x, 40646050 PMC12254498

[ref8] Azizkhani M. Saris P. E. J. Baniasadi M. (2021). An in vitro assessment of antifungal and antibacterial activity of cow, camel, ewe, and goat milk kefir and probiotic yogurt. J. Food Meas. Charact. 15, 406–415. doi: 10.1007/s11694-020-00645-4

[ref9] Bae W. Y. Min H. Shin S. L. Kim T. R. Lee H. Sohn M. . (2022). Effect of orally administered heat-treated *Lactobacillus plantarum* LM1004 on the innate immune system: a randomized, placebo-controlled, double-blind study. J. Funct. Foods 98:105293. doi: 10.1016/j.jff.2022.105293

[ref10] Bagdat E. S. Akman P. K. Kutlu G. Tornuk F. (2024). Optimization of spray-drying process parameters for microencapsulation of three probiotic lactic acid bacteria selected by their high viability rate in sucrose and fructose levels and high temperatures. Syst. Microbiol. Biomanuf. 4, 687–698. doi: 10.1007/s43393-023-00210-2

[ref11] Balamurugan S. Gemmell C. Lau A. T. Y. Arvaj L. Strange P. Gao A. . (2020). High pressure processing during drying of fermented sausages can enhance safety and reduce time required to produce a dry fermented product. Food Control 113:107224. doi: 10.1016/j.foodcont.2020.107224

[ref12] Barajas-Álvarez P. González-Ávila M. Espinosa-Andrews H. (2022). Microencapsulation of *Lactobacillus rhamnosus* HN001 by spray drying and its evaluation under gastrointestinal and storage conditions. LWT 153:112485. doi: 10.1016/j.lwt.2021.112485

[ref13] Bayram O. Y. Kinik O. Büyükkileci C. (2024). Antimicrobial activity and sensory acceptability of probiotic-strained (Torba) yogurt with medicinal and aromatic plants. S. Afr. J. Bot. 174, 218–227. doi: 10.1016/j.sajb.2024.08.053

[ref9002] Bevilacqua A. Speranza B. Campaniello D. Racioppo A. Accettulli A. De Santis A. . (2024). Effect of ultrasound-attenuation on technological and functional properties of two strains of Lactiplantibacillus plantarum isolated from table olives. Ultrasonics Sonochemistry, 110, 107057. 39236443 10.1016/j.ultsonch.2024.107057 PMC11404055

[ref14] Betoret E. Calabuig-Jiménez L. A. U. R. A. Patrignani F. Lanciotti R. Dalla Rosa M. (2017). Effect of high pressure processing and trehalose addition on functional properties of mandarin juice enriched with probiotic microorganisms. LWT-Food Sci. Technol. 85, 418–422. doi: 10.1016/j.lwt.2016.10.036

[ref15] Biçer Y. Turkal G. Sönmez G. Telli A. E. Bayir T. Çulha M. H. . (2024). Production of yoghurt from kefir beverage: analysis of fermentation kinetics, volatile organic compounds, texture, and microbial characteristics. Int. Dairy J. 158:106039. doi: 10.1016/j.idairyj.2024.106039

[ref16] Bolívar-Jacobo N. A. Reyes-Villagrana R. A. Espino-Solís G. P. Rentería-Monterrubio A. L. Arévalos-Sánchez M. M. Sánchez-Vega R. . (2023). The effects of a high-intensity ultrasound on the fermentative activity and kinetic growth of *Lactobacillus acidophilus* and *Lactobacillus helveticus*. Fermentation 9:356. doi: 10.3390/fermentation9040356

[ref17] Bommasamudram J. Muthu A. Devappa S. (2022). Effect of sub-lethal heat stress on viability of *Lacticaseibacillus casei* N in spray-dried powders. LWT 155:112904. doi: 10.1016/j.lwt.2021.112904

[ref18] Brito L. M. Costa G. A. Reis P. C. Guimarães J. T. Ramos G. L. Cruz A. G. . (2024). Impact of high-intensity ultrasound on fermentation, viability and predictive growth of lactic acid cultures: a study with conventional and probiotic fermented milks. J. Food Eng. 371:111990. doi: 10.1016/j.jfoodeng.2024.111990

[ref19] Călinoiu L. F. Vodnar D. C. Precup G. (2016). The probiotic bacteria viability under different conditions. Bull. UASVM Food Sci. Technol. 73, 55–60. doi: 10.15835/buasvmcn-fst:12448

[ref9003] Camelo-Silva C. Verruck S. Ambrosi A. Di Luccio M. (2022). Innovation and Trends in Probiotic Microencapsulation by Emulsification Techniques. Food Engineering Reviews, 14, 462–490. doi: 10.1007/s12393-022-09315-1

[ref20] Chaikham P. Yutsapremanon S. Baipong S. Jung Y. H. Zhang W. Suriyarak S. . (2025). High pressure processing of sweet corn milk yogurt: a plant-based probiotic carrier with enhanced stability. Food Chem. Adv. 9:101174. doi: 10.1016/j.focha.2025.101174

[ref21] Chanos P. Warncke M. C. Ehrmann M. A. Hertel C. (2020). Application of mild pulsed electric fields on starter culture accelerates yogurt fermentation. Eur. Food Res. Technol. 246, 621–630. doi: 10.1007/s00217-020-03428-9

[ref22] Chung I. C. OuYang C. N. Yuan S. N. Lin H. C. Huang K. Y. Wu P. S. . (2019). Pretreatment with a heat-killed probiotic modulates the NLRP3 inflammasome and attenuates colitis-associated colorectal cancer in mice. Nutrients 11:516. doi: 10.3390/nu11030516, 30823406 PMC6471765

[ref23] da Silva L. A. de Vasconcelos B. S. Moreira D. K. T. Rezende R. P. Romano C. C. Dos Santos T. F. (2023). Cupuassu juice fermentation by *Lactiplantibacillus plantarum* Lp62 produces an antioxidant enriched probiotic beverage. Lett. Appl. Microbiol. 76:ovad032. doi: 10.1093/lambio/ovad032, 36881723

[ref24] de Assis B. B. T. Cabral L. Silva F. A. de Araújo Bezerra J. Noronha M. F. Vidal H. . (2025). Fermentation of Amazonian fruit pulp (bacaba) with distinct probiotics: impacts on chemical composition, bioaccessibility, and effects on human intestinal microbiota. Food Res. Int. 209:116326. doi: 10.1016/j.foodres.2025.116326 40253160

[ref25] de Oliveira P. M. Leite Júnior B. R. D. C. Martins E. M. F. Martins M. L. Vieira É. N. R. de Barros F. A. R. . (2021). Mango and carrot mixed juice: a new matrix for the vehicle of probiotic lactobacilli. J. Food Sci. Technol. 58, 98–109. doi: 10.1007/s13197-020-04518-y, 33505055 PMC7813915

[ref26] de Sousa Melo D. da Silva L. L. Martins P. M. M. Buttrós V. H. de Souza L. R. Boas E. V. . (2025). Taro (*Colocasia esculenta*) extract fermented by a probiotic co-culture as a wall material for microencapsulation of *Lactiplantibacillus plantarum* and functional food development. Food Biosci.:108056. doi: 10.1016/j.fbio.2025.108056

[ref27] Delgado K. Vieira C. Dammak I. Frasão B. Brígida A. Costa M. . (2020). Different ultrasound exposure times influence the physicochemical and microbial quality properties in probiotic goat milk yogurt. Molecules 25:4638. doi: 10.3390/molecules25204638, 33053748 PMC7587201

[ref28] Demir E. Tappi S. Dymek K. Rocculi P. Gómez Galindo F. (2023). Reversible electroporation caused by pulsed electric field - opportunities and challenges for the food sector. Trends Food Sci. Technol. 139:104120. doi: 10.1016/j.tifs.2023.104120

[ref29] Dimitrovski D. Dimitrovska-Vetadjoka M. Hristov H. Doneva-Shapceska D. (2021). Developing probiotic pumpkin juice by fermentation with commercial probiotic strain *Lactobacillus casei* 431. J. Food Process. Preserv. 45:e15245. doi: 10.1111/jfpp.15245

[ref30] Dong X. Y. Xiu Z. L. Li S. Hou Y. M. Zhang D. J. Ren C. S. (2010). Dielectric barrier discharge plasma as a novel approach for improving 1,3-propanediol production in *Klebsiella pneumoniae*. Biotechnol. Lett. 32, 1245–1250. doi: 10.1007/s10529-010-0284-y, 20431910

[ref31] Dong X. Yuan Y. Tang Q. Dou S. Di L. Zhang X. (2014). Parameter optimization for enhancement of ethanol yield by atmospheric pressure DBD-treated *Saccharomyces cerevisiae*. Plasma Sci. Technol. 16, 73–76. doi: 10.1088/1009-0630/16/1/16

[ref32] dos Santos Rocha C. Magnani M. Klososki S. J. Marcolino V. A. dos Santos Lima M. de Freitas M. Q. . (2023). High-intensity ultrasound influences the probiotic fermentation of Baru almond beverages and impacts the bioaccessibility of phenolics and fatty acids, sensory properties, and *in vitro* biological activity. Food Res. Int. 173:113372. doi: 10.1016/j.foodres.2023.113372 37803712

[ref33] Duysburgh C. Roume H. Koper J. E. Vandekerckhove P. Duthoy S. Kemperman R. . (2025). Supplementation with probiotic/carbon source mixtures in-creased short-chain fatty acid production in the mucosal simulator of the human intestinal microbial ecosystem (M-SHIME®) model. LWT:118774. doi: 10.1016/j.lwt.2025.118774

[ref34] El Kantar S. Boussetta N. Lebovka N. Foucart F. Rajha H. N. Maroun R. G. . (2018). Pulsed electric field treatment of citrus fruits: improvement of juice and polyphenols extraction. Innov. Food Sci. Emerg. Technol. 46, 153–161. doi: 10.1016/j.ifset.2017.09.024

[ref35] Ester B. Noelia B. Laura C. J. Francesca P. Cristina B. Rosalba L. (2019). Probiotic survival and in vitro digestion of *L. salivarius* spp. *salivarius* encapsulated by high homogenization pressures and incorporated into a fruit matrix. LWT 111, 883–888. doi: 10.1016/j.lwt.2019.05.088

[ref36] Fan Y. Shang Y. Li F. Liu J. Wang D. Zhang Y. . (2024). Effects of cholesterol-lowering probiotic fermentation on the active components and in vitro hypolipidemic activity of sea buckthorn juice. J. Food Sci. 89, 6308–6320. doi: 10.1111/1750-3841.17301, 39223756

[ref37] FAO/WHO, Food and Agriculture Organization of the United Nations, and World Health Organization (2002). Guidelines for the Evaluation of Probiotics in Food: Report of a Joint FAO/WHO Working Group. Rome, Italy: FAO.

[ref38] Feng Y. Yang T. Zhang Y. Zhang A. Gai L. Niu D. (2022). Potential applications of pulsed electric field in the fermented wine industry. Front. Nutr. 9, 1048632. doi: 10.3389/fnut.2022.1048632, 36407532 PMC9668251

[ref39] Fredua-Agyeman M. Larbi E. A. (2025). Inaccurate labelling practices in probiotic products: a regulatory shortfall in Accra, Ghana. PLoS One 20:e0322194. doi: 10.1371/journal.pone.0322194, 40435307 PMC12118974

[ref40] Frontuto D. Carullo D. Harrison S. M. Brunton N. P. Ferrari G. Lyng J. G. . (2019). Optimization of pulsed electric fields-assisted extraction of polyphenols from potato peels using response surface methodology. Food Bioprocess Technol. 12, 1708–1720. doi: 10.1007/s11947-019-02320-z

[ref41] Galdeano C. M. Perdigon G. (2004). Role of viability of probiotic strains in their persistence in the gut and in mucosal immune stimulation. J. Appl. Microbiol. 97, 673–681. doi: 10.1111/j.1365-2672.2004.02353.x, 15357716

[ref42] Ghasemi L. Nouri L. Mohammadi Nafchi A. Al-Hassan A. A. (2022). The effects of encapsulated probiotic bacteria on the physicochemical properties, staling, and viability of probiotic bacteria in gluten-free bread. J. Food Process. Preserv. 46:e16359. doi: 10.1111/jfpp.16359

[ref43] Gholamhosseinpour A. Hashemi S. M. B. (2019). Ultrasound pretreatment of fermented milk containing probiotic *Lactobacillus plantarum* AF1: carbohydrate metabolism and antioxidant activity. J. Food Process Eng. 42:e12930. doi: 10.1111/jfpe.12930

[ref44] Gholian M. Babaei A. Mortazavian S. A. M. Koushki V. (2023). Evaluating physicho-chemical and sensory properties of grape juice fortified with paraprobiotic *Lactobacillus gasseri* during storage. J. Food Sci. Technol. 20, 49–60. doi: 10.22034/FSCT.20.141.49

[ref45] Gibson G. R. Roberfroid M. B. (1995). Dietary modulation of the human colonic microbiota: introducing the concept of prebiotics. J. Nutr. 125, 1401–1412. doi: 10.1093/jn/125.6.1401, 7782892

[ref46] Giordano I. Abuqwider J. Altamimi M. Di Monaco R. Puleo S. Mauriello G. (2022). Application of ultrasound and microencapsulation on *Limosilactobacillus reuteri* DSM 17938 as a metabolic attenuation strategy for tomato juice probiotication. Heliyon 8:e10969. doi: 10.1016/j.heliyon.2022.e10969, 36254285 PMC9568839

[ref47] Gökmen G. G. Sarıyıldız S. Cholakov R. Nalbantsoy A. Baler B. Aslan E. . (2024). A novel *Lactiplantibacillus plantarum* strain: probiotic properties and optimization of the growth conditions by response surface methodology. World J. Microbiol. Biotechnol. 40:66. doi: 10.1007/s11274-023-03862-3 38194015 PMC10776492

[ref48] Goktas H. Dikmen H. Bekiroglu H. Cebi N. Dertli E. Sagdic O. (2022). Characteristics of functional ice cream produced with probiotic *Saccharomyces boulardii* in combination with *Lactobacillus rhamnosus* GG. LWT 153:112489. doi: 10.1016/j.lwt.2021.112489

[ref49] Gong P. Lin K. Zhang J. Han X. Lyu L. Yi H. . (2020). Enhancing spray drying tolerance of *Lactobacillus bulgaricus* by intracellular trehalose delivery via electroporation. Food Res. Int. 127:108725. doi: 10.1016/j.foodres.2019.108725, 31882105

[ref50] Gurram S. Jha D. K. Shah D. S. Kshirsagar M. M. Amin P. D. (2021). Insights on the critical parameters affecting the probiotic viability during stabilization process and formulation development. AAPS PharmSciTech 22:156. doi: 10.1208/s12249-021-02024-8, 34008083

[ref51] Hansen S. J. Tang P. Kiefer A. Galles K. Wong C. Morovic W. (2020). Droplet digital PCR is an improved alternative method for high-quality enumeration of viable probiotic strains. Front. Microbiol. 10:3025. doi: 10.3389/fmicb.2019.03025, 32038522 PMC6987037

[ref52] Hao F. Fu N. Ndiaye H. Woo M. W. Jeantet R. Chen X. D. (2021). Thermotolerance, survival, and stability of lactic acid bacteria after spray drying as affected by the increase of growth temperature. Food Bioprocess Technol. 14, 120–132. doi: 10.1007/s11947-020-02571-1

[ref53] Harahap N. I. P. Munir E. Hutahaean S. (2023). Immunomodulatory effects of probiotics isolated from traditional fermented foods and beverages of Sumatra (Indonesia) and synbiotics in mice. Biodiversitas 24, 1157. doi: 10.13057/biodiv/d240256

[ref54] Haro-González J. N. Velásquez-Reyes D. D. C. (2025). Sensory impact of probiotic incorporation in food matrices: a systematic review. Crit. Rev. Food Sci. Nutr. 66, 1–22. doi: 10.1080/10408398.2025.2593583 41363860

[ref55] Hasgucmen C. K. Sengun I. Y. (2020). Viability of probiotic strain *Lactobacillus rhamnosus* and its impact on sensory properties of cheesecake during storage at −20°C and 4°C. LWT 134:109967. doi: 10.1016/j.lwt.2020.109967

[ref56] Herrera-Ponce A. L. Salmeron-Ochoa I. Rodriguez-Figueroa J. C. Santellano-Estrada E. Garcia-Galicia I. A. Alarcon-Rojo A. D. (2022). High-intensity ultrasound as pre-treatment in the development of fermented whey and oat beverages: effect on the fermentation, antioxidant activity and consumer acceptance. J. Food Sci. Technol. 59, 796–804. doi: 10.1007/s13197-021-05074-9, 35153316 PMC8814079

[ref57] Hill C. Guarner F. Reid G. Gibson G. R. Merenstein D. J. Pot B. . (2014). The international scientific Association for Probiotics and Prebiotics consensus statement on the scope and appropriate use of the term probiotic. Nat. Rev. Gastroenterol. Hepatol. 11, 506–514. doi: 10.1038/nrgastro.2014.66, 24912386

[ref58] Hong G. H. Lee S. Y. Kim I. A. Suk J. Baeg C. Kim J. Y. . (2024). Effect of heat-treated *Lactiplantibacillus plantarum* nF1 on the immune system including natural killer cell activity: a randomized, placebo-controlled, double-blind study. Nutrients 16:1339. doi: 10.3390/nu16091339 38732587 PMC11085399

[ref59] Iglesias M. B. Viñas I. Colás-Medà P. Collazo C. Serrano J. C. E. Abadias M. (2017). Adhesion and invasion of *listeria monocytogenes* and interaction with *Lactobacillus rhamnosus* GG after habituation on fresh-cut pear. J. Funct. Foods 34, 453–460. doi: 10.1016/j.jff.2017.05.011

[ref60] ISAPP (2022). Decoding a probiotic product label. Available online at: https://isappscience.org/decoding-a-probiotic-product-label/ (Accessed January 12, 2025).

[ref61] Jara-Quijada E. Pérez-Won M. Tabilo-Munizaga G. González-Cavieres L. Palma-Acevedo A. Herrera-Lavados C. . (2024). Polyphenol extraction of green tea through pulsed electric field: yield improvement and environmental assessment. Food Bioprocess Technol. 17, 2718–2734. doi: 10.1007/s11947-023-03286-9

[ref62] Jeong S. H. Jung Y. M. Kim S. Kim J. H. Yeo H. Lee D. U. (2023). Tenderization of beef semitendinosus muscle by pulsed electric field treatment with a direct contact chamber and its impact on proteolysis and physicochemical properties. Foods 12:430. doi: 10.3390/foods12030430, 36765959 PMC9913947

[ref63] Jiang Z. Li M. McClements D. J. Liu X. Liu F. (2022). Recent advances in the design and fabrication of probiotic delivery systems to target intestinal inflammation. Food Hydrocoll. 125:107438. doi: 10.1016/j.foodres.2025.116826

[ref64] Jiang Y. H. Li Y. Y. Xin W. G. Song J. J. Wang C. Suo H. Y. (2025). Protective effect of yak yogurt derived *Lactiplantibacillus plantarum* dacnjs22 fermented milk on dss-induced colitis: insights from in vitro and in vivo studies. Food Res. Int. 217:116826. doi: 10.1016/j.foodres.2025.116826, 40597533

[ref65] Kanafusa S. Uhlig E. Uemura K. Galindo F. G. Håkansson Å. (2021). The effect of nanosecond pulsed electric field on the production of metabolites from lactic acid bacteria in fermented watermelon juice. Innov. Food Sci. Emerg. Technol. 72:102749. doi: 10.1016/j.ifset.2021.102749

[ref66] Kang J. Lee J. J. Cho J. H. Choe J. Kyoung H. Kim S. H. . (2021). Effects of dietary inactivated probiotics on growth performance and immune responses of weaned pigs. J. Anim. Sci. Technol. 63, 520–530. doi: 10.5187/jast.2021.e44, 34189502 PMC8203999

[ref67] Kardooni Z. Alizadeh Behbahani B. Jooyandeh H. Noshad M. (2023). Probiotic viability, physicochemical, and sensory properties of probiotic orange juice. J. Food Meas. Charact. 17, 1817–1822. doi: 10.1007/s11694-022-01771-x

[ref68] Kemsawasd V. Chaikham P. (2020). Effects of frozen storage on viability of probiotics and antioxidant capacities of synbiotic riceberry and sesame-riceberry milk ice creams. Curr. Res. Nutr. Food Sci. J. 8, 107–121. doi: 10.12944/crnfsj.8.1.10

[ref69] Kiefer A. Tang P. Arndt S. Fallico V. Wong C. (2020). Optimization of viability treatment essential for accurate droplet digital PCR enumeration of probiotics. Front. Microbiol. 11:1811. doi: 10.3389/fmicb.2020.01811, 32849418 PMC7399075

[ref70] Kokwar M. A. Arya S. S. Bhat M. S. (2022). A cereal-based nondairy probiotic functional beverage: an insight into the improvement in quality characteristics, sensory profile, and shelf-life. J. Food Process. Preserv. 46:e16147. doi: 10.1111/jfpp.16147

[ref71] Küçükgöz K. Kruk M. Kołożyn-Krajewska D. Trząskowska M. (2024). Investigating the probiotic potential of vegan puree mixture: viability during simulated digestion and bioactive compound bioaccessibility. Nutrients 16:561. doi: 10.3390/nu16040561, 38398885 PMC10893087

[ref72] Lee A. Lee Y. J. Yoo H. J. Kim M. Chang Y. Lee D. S. . (2017). Consumption of dairy yogurt containing *Lactobacillus paracasei* ssp. *paracasei*, *Bifidobacterium animalis* ssp. *lactis* and heat-treated *Lactobacillus plantarum* improves immune function including natural killer cell activity. Nutrients 9:558. doi: 10.3390/nu9060558, 28561762 PMC5490537

[ref73] Lin S. P. Kuo T. C. Wang H. T. Ting Y. Hsieh C. W. Chen Y. K. . (2020). Enhanced bioethanol production using atmospheric cold plasma-assisted detoxification of sugarcane bagasse hydrolysate. Bioresour. Technol. 313:123704. doi: 10.1016/j.biortech.2020.123704, 32590306

[ref9006] Liu W. S. Yang C. Y. Fang T. J. (2018). Strategic ultrasound-induced stress response of lactic acid bacteria on enhancement of β-glucosidase activity for bioconversion of isoflavones in soymilk. Journal of Microbiological Methods, 148, 145–150. 29656125 10.1016/j.mimet.2018.04.006

[ref74] Liu Y. Mijiti M. Xu Z. Abulikemu B. (2024). Effect of a combination of probiotics on the flavor profiling and biogenic amines of composite fermented mutton sausages. Food Biosci. 61:104835. doi: 10.1016/j.fbio.2024.104835

[ref75] Liu Y. Wang J. Wu C. (2022). Modulation of gut microbiota and immune system by probiotics, pre-biotics, and post-biotics. Front. Nutr. 8:634897. doi: 10.3389/fnut.2021.634897, 35047537 PMC8761849

[ref76] Ma L. Chang D. Lv J. Yan Y. Zheng A. Wang X. . (2026). *Lactiplantibacillus plantarum* JLAU103 fermented walnut-milk dual-protein yogurt: physicochemical property, volatile flavor profile, antioxidant activity, and isolation of neuroprotective peptides targeting Aβ1-42 oligomers. Food Biosci. 76:108260. doi: 10.1016/j.fbio.2026.108260

[ref77] Ma J. Wang Y. Zhao M. Tong P. Lv L. Gao Z. . (2023). High hydrostatic pressure treatments improved properties of fermentation of apple juice accompanied by higher reserved *Lactobacillus plantarum*. Foods 12:441. doi: 10.3390/foods12030441 36765970 PMC9913918

[ref78] Machado M. Sousa S. C. Rodríguez-Alcalá L. M. Pintado M. Gomes A. M. (2023). Functional lipid enriched probiotic cheese: gastrointestinal stability and potential health benefits. Int. Dairy J. 144:105700. doi: 10.1016/j.idairyj.2023.105700

[ref79] Mahmoud M. Abdallah N. A. El-Shafei K. Tawfik N. F. El-Sayed H. S. (2020). Survivability of alginate-microencapsulated *Lactobacillus plantarum* during storage, simulated food processing and gastrointestinal conditions. Heliyon 6:e03541. doi: 10.1016/j.heliyon.2020.e03541, 32190759 PMC7068628

[ref80] Mahmud S. Khan S. Khan M. R. Islam J. Sarker U. K. Hasan G. M. M. A. . (2022). Viability and stability of microencapsulated probiotic bacteria by freeze-drying under in vitro gastrointestinal conditions. J. Food Process. Preserv. 46:e17123. doi: 10.1111/jfpp.17123

[ref81] Malos I. G. Pasarin D. Ghizdareanu A. I. Frunzareanu B. (2025). A promising approach for the food industry: enhancing probiotic viability through microencapsulated synbiotics. Microorganisms 13:336. doi: 10.3390/microorganisms13020336, 40005703 PMC11858381

[ref82] Marco M. L. Sanders M. E. Gänzle M. Arrieta M. C. Cotter P. D. De Vuyst L. . (2021). The international scientific Association for Probiotics and Prebiotics (ISAPP) consensus statement on fermented foods. Nat. Rev. Gastroenterol. Hepatol. 18, 196–208. doi: 10.1038/s41575-020-00390-5, 33398112 PMC7925329

[ref83] Masoumi S. J. Mehrabani D. Saberifiroozi M. Fattahi M. R. Moradi F. Najafi M. (2021). The effect of yogurt fortified with *Lactobacillus acidophilus* and *Bifidobacterium* sp. probiotic in patients with lactose intolerance. Food Sci. Nutr. 9, 1704–1711. doi: 10.1002/fsn3.2145, 33747481 PMC7958570

[ref84] Maxan M. E. Moens F. Marzorati M. Mohamad M. Yue C. Said J. . (2026). A multi-strain probiotic modulates gut microbiome composition, intestinal barrier integrity and inflammation in a multi-compartmental in vitro gut model of decompensated alcohol-related advanced chronic liver disease. Int. J. Pharm., 693:126678. doi: 10.1016/j.ijpharm.2026.126678 41698451

[ref85] McGillin M. R. deRiancho D. L. DeMarsh T. A. Hsu E. D. Alcaine S. D. (2022). Selective survival of protective cultures during high-pressure processing by leveraging freeze-drying and encapsulation. Foods 11:2465. doi: 10.3390/foods11162465, 36010466 PMC9407356

[ref86] Meena L. Malini B. Byresh T. S. Sunil C. K. Rawson A. Venkatachalapathy N. (2024). Ultrasound as a pre-treatment in millet-based probiotic beverage: it’s effect on fermentation kinetics and beverage quality. Food Chem Adv 4:100631. doi: 10.1016/J.FOCHA.2024.100631

[ref87] Mehta D. Chaturvedi K. Sidana A. Shivhare U. S. Yadav S. K. (2022). Processing treatment of atmospheric and vacuum-cold plasma improved physical properties, glucose diffusion and fermentability of dietary fibers extracted from de-oiled rice and corn bran. Bioact. Carbohydr. Diet. Fibre 28:100326. doi: 10.1016/j.bcdf.2022.100326, 38826717

[ref9004] Meybodi N. M. Mortazavian A. M. Arab M. Nematollahi A. (2020). Probiotic viability in yoghurt: A review of influential factors. International Dairy Journal, 109, 104793.

[ref88] Miranda-Mejía G. A. Martín del Campo-Barba S. T. Arredondo-Ochoa T. Tejada-Ortigoza V. la Peña M. M. de. (2024). Low-intensity pulsed electric fields pre-treatment on yogurt starter culture: effects on fermentation time and quality attributes. Innov. Food Sci. Emerg. Technol., 95:103708. doi: 10.1016/J.IFSET.2024.103708

[ref89] Mis-Solval K. E. Jiang N. Yuan M. Joo K. H. Cavender G. A. (2019). The effect of the ultra-high-pressure homogenization of protein encapsulants on the survivability of probiotic cultures after spray drying. Foods 8:689. doi: 10.3390/foods8120689, 31861054 PMC6963204

[ref90] Mizuta A. G. de Menezes J. L. da Silva L. A. Marcolino V. A. Barao C. E. Pimentel T. C. . (2023). High-intensity ultrasound reduces fermentation time and improves textural properties, antioxidant activity and probiotic survival in fermented probiotic strawberry drink. Int. J. Food Sci. Technol. 58, 194–204. doi: 10.1111/ijfs.16187

[ref91] Moens L. G. Huang W. Van Loey A. M. Hendrickx M. E. (2021). Effect of pulsed electric field and mild thermal processing on texture-related pectin properties to better understand carrot (*Daucus carota*) texture changes during subsequent cooking. Innov. Food Sci. Emerg. Technol. 70, 102700. doi: 10.1016/j.ifset.2021.102700

[ref92] Mokhtari S. Jafari S. M. Khomeiri M. (2019). Survival of encapsulated probiotics in pasteurized grape juice and evaluation of their properties during storage. Food Sci. Technol. Int. 25, 120–129. doi: 10.1177/1082013218801113, 30235945

[ref93] Morais A. T. B. Morais S. T. B. Feitor J. F. Cavalcante K. N. Catunda L. G. S. Walkling-Ribeiro M. . (2023). Physico-chemical and structural modifications of caseins in micellar casein isolate induced by pulsed electric field. Innov. Food Sci. Emerg. Technol. 89:103476. doi: 10.1016/J.IFSET.2023.103476

[ref94] Moreno A. Meca G. Esteban-Torres M. Escrivá L. (2025). Protective effect on intestinal barrier and cell viability of probiotic *Lacticaseibacillus* strain after simulated gastrointestinal digestion with dairy and vegetal products. Food Biosci. 73:107583. doi: 10.1016/j.fbio.2025.107583, 38826717

[ref9005] Mostafa H. S. Ali M. R. Mohamed R. M. (2020). Production of a novel probiotic date juice with anti-proliferative activity against Hep-2 cancer cells. Food Science and Technology, 41(suppl 1), 105–115.

[ref95] Moura F. T. d. Silva S. d. M. Sousa F. d. A. R. d. M. Santos K. M. Bastos T. M. d. R. Araújo J. (2024). Application of pulsed electric field in reducing internal browning and maintaining the functional potential of ‘Pérola’ pineapple. Rev. Cienc. Agron. 55:e20217851. doi: 10.5935/1806-6690.20240027

[ref96] Munekata P. E. Alcántara C. Žugčić T. Abdelkebir R. Collado M. C. García-Pérez J. V. . (2020). Impact of ultrasound-assisted extraction and solvent composition on bioactive compounds and in vitro biological activities of thyme and rosemary. Food Res. Int. 134:109242. doi: 10.1016/j.foodres.2020.109242 32517919

[ref9012] Müller W. A. Ferreira Marczak L. D. Sarkis J. R. (2020). Microbial inactivation by ohmic heating:Literature review and influence of different process variables. Trends in Food Science & Technology, 99, 650–659. doi: 10.1016/j.tifs.2020.03.021

[ref9007] Mukherjee A. Gómez-Sala B. O\u0027Connor E. M. Kenny J. G. Cotter P. D. (2022). Global regulatory frameworks for fermented foods: a review. Frontiers in nutrition, 9, 902642. 35719144 10.3389/fnut.2022.902642 PMC9198641

[ref97] Niamah A. K. Al-Fekaiki D. F. Thyab Gddoa Al-Sahlany S. Verma D. K. Patel A. R. Singh S. (2023). Investigating the effect of addition of probiotic microorganisms (bacteria or yeast) to yoghurt on the viability and volatile aromatic profiles. J. Food Meas. Charact. 17, 5463–5473. doi: 10.1007/s11694-023-02056-7

[ref98] Ocampo-Zaragoza A. A. Grajales-Lagunes A. Ovando-Chacón S. L. Luján-Hidalgo M. C. Manuel Ruíz-Valdiviezo V. Ruiz-Cabrera M. A. . (2026). Immunomodulatory effects of *Lactiplantibacillus paraplantarum* BAL-28-ITTG paraprobiotics and postbiotics. Lett. Appl. Microbiol. 79:ovag033. doi: 10.1093/lambio/ovag033 41811992

[ref99] Olivares A. Soto C. Caballero E. Altamirano C. (2019). Survival of microencapsulated *Lactobacillus casei* (prepared by vibration technology) in fruit juice during cold storage. Electron. J. Biotechnol. 42, 42–48. doi: 10.1016/j.ejbt.2019.10.002

[ref100] Oliveira P. M. D. Leite Júnior B. R. D. C. Martins E. M. F. Cristianini M. Martins M. L. Vieira É. N. R. . (2020). Impact of high pressure and thermal processing on probiotic mixed mango and carrot juices. J. Food Process. Preserv. 44:e14530. doi: 10.1111/jfpp.14530

[ref101] Pandey P. Mettu S. Mishra H. N. Ashokkumar M. Martin G. J. (2021). Multilayer co-encapsulation of probiotics and γ-amino butyric acid (GABA) using ultrasound for functional food applications. LWT 146:111432. doi: 10.1016/j.lwt.2021.111432

[ref102] Panel E. B. Koutsoumanis K. Allende A. Álvarez-Ordóñez A. Bolton D. Bover-Cid S. . (2022). Updated list of QPS-recommended biological agents for safety risk assessments carried out by EFSA Zenodo Parma Italy. doi: 10.5281/ZENODO.6902983

[ref103] Pankiewicz U. Góral M. Kozłowicz K. Góral D. (2020). Application of pulsed electric field in production of ice cream enriched with probiotic bacteria (*L. rhamnosus* B 442) containing intracellular calcium ions. J. Food Eng. 275:109876. doi: 10.1016/J.JFOODENG.2019.109876

[ref104] Papakonstantinou E. Zacharodimos N. Georgiopoulos G. Athanasaki C. Bothou D. L. Tsitsou S. . (2024). Two-month consumption of orange juice enriched with vitamin D3 and probiotics decreases body weight, insulin resistance, blood lipids, and arterial blood pressure in high-cardiometabolic-risk patients on a westernized type diet: results from a randomized clinical trial. Nutrients 16:1331. doi: 10.3390/nu16091331, 38732578 PMC11085203

[ref105] Pappas V. M. Lakka A. Palaiogiannis D. Bozinou E. Ntourtoglou G. Batra G. . (2021). Use of pulsed electric field as a low-temperature and high performance “green” extraction technique for the recovery of high added value compounds from olive leaves. Beverages 7:45. doi: 10.3390/beverages7030045

[ref106] Parvarei M. M. Fazeli M. R. Mortazavian A. M. Nezhad S. S. Mortazavi S. A. Golabchifar A. A. . (2021). Comparative effects of probiotic and paraprobiotic addition on microbiological, biochemical and physical properties of yogurt. Food Res. Int. 140:110030. doi: 10.1016/j.foodres.2020.110030 33648258

[ref107] Pereira R. N. Teixeira J. A. Vicente A. A. Cappato L. P. da Silva Ferreira M. V. da Silva Rocha R. . (2018). Ohmic heating for the dairy industry: a potential technology to develop probiotic dairy foods in association with modifications of whey protein structure. Curr. Opin. Food Sci. 22, 95–101. doi: 10.1016/j.cofs.2018.01.014

[ref9008] Peng K. Koubaa M. Bals O. Vorobiev E. (2020). Effect of pulsed electric fields on the growth and acidification kinetics of Lactobacillus delbrueckii Subsp. bulgaricus. Foods, 9, 1146. 32825249 10.3390/foods9091146 PMC7555770

[ref108] Pina-Pérez M. C. Ricós-Muñoz N. López-Suárez E. K. Esteve C. Maicas S. Beyrer M. (2024). Impact of cold atmospheric pressure plasma (CAPP) treatments on the prebiotic potential of *Arthrospira platensis* (Spirulina). Algal Res. 78:103432. doi: 10.1016/j.algal.2024.103432

[ref109] Potoroko I. Kalinina I. Botvinnikova V. Krasulya O. Fatkullin R. Bagale U. . (2018). Ultrasound effects based on simulation of milk processing properties. Ultrason. Sonochem. 48, 463–472. doi: 10.1016/j.ultsonch.2018.06.019, 30080573

[ref110] Prangthip P. Chaikham P. (2025). Probiotic-enriched Maoberry juice powder: effects of carrier dextrose equivalent on physicochemical characteristics and gut microbiome. Appl. Food Res. 5:101514. doi: 10.1016/j.afres.2025.101514

[ref111] Qayyum N. Haoyue H. Ismael M. Yantin Q. Lü X. (2024). In vitro assessment of antioxidant, antidiabetic, and cholesterol-modulating abilities of lactic acid bacteria: implications for metabolic health and functional foods. Food Biosci. 59:103952. doi: 10.1016/j.fbio.2024.103952

[ref112] Qiao W. Qiao Y. Liu F. Zhang Y. Li R. Wu Z. . (2020). Engineering *Lactococcus lactis* as a multi-stress tolerant biosynthetic chassis by deleting the prophage-related fragment. Microb. Cell Factories 19:225. doi: 10.1186/s12934-020-01487-x PMC7727215 33298073

[ref113] Racioppo A. Corbo M. R. Piccoli C. Sinigaglia M. Speranza B. Bevilacqua A. (2017). Ultrasound attenuation of lactobacilli and bifidobacteria: effect on some technological and probiotic properties. Int. J. Food Microbiol. 243, 78–83. doi: 10.1016/j.ijfoodmicro.2016.12.011, 28038333

[ref114] Racioppo A. Speranza B. Altieri C. Sinigaglia M. Corbo M. R. Bevilacqua A. (2023). Ultrasound can increase biofilm formation by *Lactiplantibacillus plantarum* and *Bifidobacterium* spp. Front. Microbiol. 14:1094671. doi: 10.3389/FMICB.2023.1094671, 36950165 PMC10025361

[ref115] Rafiq S. Huma N. Gulzar N. Murtaza M. A. Hussain I. (2018a). Effect of cheddar cheese peptide extracts on growth inhibition, cell cycle arrest and apoptosis induction in human lung cancer (H-1299) cell line. Int. J. Dairy Technol. 71, 975–980. doi: 10.1111/1471-0307.12533

[ref116] Rafiq S. Huma N. Rakariyatham K. Hussain I. Gulzar N. Hayat I. (2018b). Anti-inflammatory and anticancer activities of water-soluble peptide extracts of buffalo and cow milk cheddar cheeses. Int. J. Dairy Technol. 71, 432–438. doi: 10.1111/1471-0307.12483

[ref117] Rajam R. Subramanian P. (2022). Encapsulation of probiotics: past, present and future. Beni-Suef Univ. J. Basic Appl. Sci. 11:46. doi: 10.1186/s43088-022-00228-w

[ref118] Rezaee K. Noghabi M. S. Behzad K. Maskooki A. (2019). Effect of moderate pulsed electric field treatment on viscoelastic properties of sugar beet. Food Sci. Technol. Res. 25, 157–166. doi: 10.3136/fstr.25.157

[ref119] Ricci A. Parpinello G. P. Banfi B. A. Olivi F. Versari A. (2020). Preliminary study of the effects of pulsed electric field (PEF) treatments in wines obtained from early-harvested sangiovese grapes. Beverages 6:34. doi: 10.3390/beverages6020034

[ref120] Roobab U. Chen B.-R. Madni G. M. Tong Z. G. Zeng X.-A. Abdi G. . (2024). Evaluation of ultrasound and pulsed electric field combinations on the cooking losses, texture profile, and taste-related amino acids of chicken breast meat. Ultrason. Sonochem. 107:106919. doi: 10.1016/j.ultsonch.2024.106919 38795569 PMC11144803

[ref9009] Rogovski P. Cadamuro R. D. da Silva R. de Souza E. B. Bonatto C. Viancelli A. (2021). Uses of Bacteriophages as Bacterial Control Tools and Environmental Safety Indicators [Mini Review]. Frontiers in Microbiology, 12. doi: 10.3389/fmicb.2021.79313 PMC8670004 34917066

[ref121] Rosa L. S. Santos M. L. Abreu J. P. Balthazar C. F. Rocha R. S. Silva H. L. . (2020). Antiproliferative and apoptotic effects of probiotic whey dairy beverages in human prostate cell lines. Food Res. Int. 137:109450. doi: 10.1016/j.foodres.2020.109450, 33233128

[ref122] Salas-Millán J. Á. Conesa-Bueno A. Aguayo E. (2024). A novel antidiabetic lactofermented beverage from agro-industrial waste (broccoli leaves): process optimisation, phytochemical characterisation, and shelf-life through thermal treatment and high hydrostatic pressure. Food Biosci. 59:103999. doi: 10.1016/j.fbio.2024.103999

[ref123] Samaranayake C. P. Mok J. H. Heskitt B. F. Sastry S. K. (2022). Nonthermal inactivation of polyphenol oxidase in apple juice influenced by moderate electric fields: effects of periodic on-off and constant exposure electrical treatments. Innov. Food Sci. Emerg. Technol. 77:102955. doi: 10.1016/j.ifset.2022.102955

[ref124] Santos N. C. Almeida R. L. J. Monteiro S. S. de Alcântara Silva V. M. de Lima T. L. B. Saraiva M. M. T. . (2024). Ultrasound and microwaves reduce stress in probiotics during avocado drying: impact on mass transfer and cell viability. Food Biosci. 61:104655. doi: 10.1016/j.fbio.2024.104655

[ref125] Sepehr A. Aghamohammad S. Ghanavati R. Bavandpour A. K. Talebi M. Rohani M. . (2024). The inhibitory effects of the novel *Lactobacillus cocktail* on colorectal cancer development through modulating BMP signaling pathway: *in vitro* and *in vivo* study. Heliyon 10:e36554. doi: 10.1016/j.heliyon.2024.e36554, 39281652 PMC11402137

[ref126] Shi Z. Wu J. Wang X. Nie T. Zeng Q. Yuan C. . (2024). Development of Pickering water-in-oil emulsions using a dual stabilization of candelilla wax and acylated EGCG derivatives to enhance the survival of probiotics (*Lactobacillus plantarum*) powder. Food Funct. 15, 11141–11157. doi: 10.1039/D4FO01342E, 39440390

[ref127] Shokri S. Terefe N. S. Shekarforoush S. S. Hosseinzadeh S. (2021). Ultrasound-assisted fermentation for enhancing metabolic and probiotic activities of *Lactobacillus brevis*. Chem. Eng. Process. Process Intensif. 166:108470. doi: 10.1016/j.cep.2021.108470

[ref128] Siciliano R. A. Reale A. Mazzeo M. F. Morandi S. Silvetti T. Brasca M. (2021). Paraprobiotics: a new perspective for functional foods and nutraceuticals. Nutrients 13:1225. doi: 10.3390/nu13041225, 33917707 PMC8068161

[ref129] Sim S. Y. Hua X. Y. Henry C. J. (2020). A novel approach to structure plant-based yogurts using high pressure processing. Foods 9:1126. doi: 10.3390/foods9081126, 32824140 PMC7466357

[ref130] Siroli L. Braschi G. Rossi S. Gottardi D. Patrignani F. Lanciotti R. (2020). *Lactobacillus paracasei* A13 and high-pressure homogenization stress response. Microorganisms 8:439. doi: 10.3390/microorganisms8030439, 32244939 PMC7143770

[ref131] Sotelo K. A. G. Hamid N. Oey I. Pook C. Gutierrez-Maddox N. Ma Q. . (2018). Red cherries (*Prunus avium* var. Stella) processed by pulsed electric field - physical, chemical and microbiological analyses. Food Chem. 240, 926–934. doi: 10.1016/J.FOODCHEM.2017.08.017, 28946363

[ref132] Souza F. E. B. Rodrigues S. Fonteles T. V. (2025). Non-thermal technologies in food fermentation: mechanisms, benefits, and industrial perspectives for sustainable development. PRO 13:2988. doi: 10.3390/pr13092988

[ref133] Sultana M. Chan E. S. Janarthanan P. Choo W. S. (2023). Functional orange juice with *Lactobacillus casei* and tocotrienol-enriched flaxseed oil co-encapsulation: physicochemical properties, probiotic viability, oxidative stability, and sensorial acceptability. LWT 188:115388. doi: 10.1016/j.lwt.2023.115388

[ref134] Sun R. Wang Y. Lv Z. Li H. Zhang S. Dang Q. . (2024). Construction of Fu brick tea polysaccharide-cold plasma modified alginate microgels for probiotic delivery: enhancing viability and colonization. Int. J. Biol. Macromol. 268:131899. doi: 10.1016/j.ijbiomac.2024.131899, 38677703

[ref135] Swanson K. S. Gibson G. R. Hutkins R. Reimer R. A. Reid G. Verbeke K. . (2020). The international scientific Association for Probiotics and Prebiotics (ISAPP) consensus statement on the definition and scope of synbiotics. Nat. Rev. Gastroenterol. Hepatol. 17, 687–701. doi: 10.1038/s41575-020-0344-2, 32826966 PMC7581511

[ref136] Szajewska H. Vinderola G. (2024). “Current regulatory issues for the use of probiotics,” in Probiotics and Child Gastrointestinal Health: Advances in Microbiology, Infectious Diseases and Public Health Volume 19, eds. Guandalini S. Indrio F. (Cham, Switzerland: Springer International Publishing), 187–193.

[ref137] Taha A. Casanova F. Šimonis P. Stankevič V. Gomaa M. A. E. Stirkė A. (2022). Pulsed electric field: fundamentals and effects on the structural and techno-functional properties of dairy and plant proteins. Foods 11:1556. doi: 10.3390/FOODS11111556 35681305 PMC9180040

[ref138] Taverniti V. Guglielmetti S. (2011). The immunomodulatory properties of probiotic microorganisms beyond their viability (ghost probiotics: proposal of paraprobiotic concept). Genes Nutr. 6, 261–274. doi: 10.1007/s12263-011-0218-x, 21499799 PMC3145061

[ref139] Thinkohkaew K. Jonjaroen V. Niamsiri N. McClements D. J. Panya A. Suppavorasatit I. . (2025). Fabrication of synbiotic carbohydrate polymer-based microcapsules: effect of prebiotics on probiotic viability during freeze-drying, gastrointestinal transit and storage. Carbohydr. Polym. 359:123582. doi: 10.1016/j.carbpol.2025.123582, 40306787

[ref140] Thompson T. Kilders V. Widmar N. Ebner P. (2024). Consumer acceptance of bacteriophage technology for microbial control. Sci. Rep. 14:25279. doi: 10.1038/s41598-024-75721-6, 39455687 PMC11512061

[ref141] Tsevdou M. Ouli-Rousi M. Soukoulis C. Taoukis P. (2020). Impact of high-pressure process on probiotics: viability kinetics and evaluation of the quality characteristics of probiotic yoghurt. Foods 9:360. doi: 10.3390/foods9030360, 32204574 PMC7142589

[ref142] Tukel O. Sengun I. (2024). Production of probiotic fermented salami using *Lacticaseibacillus rhamnosus*, *Lactiplantibacillus plantarum*, and *Bifidobacterium lactis*. J. Food Sci. 89, 2956–2973. doi: 10.1111/1750-3841.17058, 38602050 PMC13281135

[ref9011] Vaessen E. M. den Besten H. M. Leito K. M. Schutyser M. A. (2020). Pulsed electric field pre-treatment for enhanced bacterial survival after drying: Effect of carrier matrix and strain variability. Innovative Food Science & Emerging Technologies, 66, 102515.

[ref143] Valero-Cases E. Frutos M. J. Pérez-Llamas F. (2023). Development of synbiotic vegan beverages: probiotic viability, sensory profile, consumers' acceptance and functional stability. Int. J. Food Sci. Technol. 58, 2325–2335. doi: 10.1111/ijfs.16361

[ref144] Valero-Cases E. Nuncio-Jáuregui N. Frutos M. J. (2017). Influence of fermentation with different lactic acid bacteria and *in vitro* digestion on the biotransformation of phenolic compounds in fermented pomegranate juices. J. Agric. Food Chem. 65, 6488–6496. doi: 10.1021/acs.jafc.6b04854, 28274113

[ref145] Vieira É. N. R. de Oliveira V. C. Gomes A. T. Lourenço M. T. e Paiva M. J. D. A. Santos T. C. . (2024). Perspectives of high-pressure technology in probiotic food production: a comprehensive review. Food Biosci.:105179. doi: 10.1016/j.fbio.2024.105179

[ref146] Wang L. Y. Huang Z. L. Li G. Zhao H. X. Xing X. H. Sun W. T. . (2010). Novel mutation breeding method for *Streptomyces avermitilis* using an atmospheric pressure glow discharge plasma. J. Appl. Microbiol. 108, 851–858. doi: 10.1111/j.1365-2672.2009.04483.x, 19735332

[ref147] Wang H. Tao Y. Li Y. Wu S. Li D. Liu X. . (2021). Application of ultrasonication at different microbial growth stages during apple juice fermentation by *Lactobacillus plantarum*: investigation on the metabolic response. Ultrason. Sonochem. 73:105486. doi: 10.1016/j.ultsonch.2021.105486, 33639530 PMC7921625

[ref148] Wang R. Wang L.-H. Wen Q.-H. He F. Xu F.-Y. Chen B.-R. . (2023). Combination of pulsed electric field and pH shifting improves the solubility, emulsifying, foaming of commercial soy protein isolate. Food Hydrocoll. 134:108049. doi: 10.1016/j.foodhyd.2022.108049

[ref149] Welk A. K. Mehlhose C. Daum D. Enneking U. (2025). Exploring customer segmentation for food products with additional health benefits: a case study on iron-biofortified vegetables, functional foods, and dietary supplements. Appetite 211:108004. doi: 10.1016/j.appet.2025.108004, 40194561

[ref150] Wen Q. Chen X. Xu M. Liu R. Lian W. Ma Y. . (2024). Selection and characterization of spontaneous phage-resistant mutant of *Limosilactobacillus fermentum*. Int. J. Food Microbiol. 423:110833. doi: 10.1016/j.ijfoodmicro.2024.110833, 39079450

[ref9021] Wendel U. (2022). Assessing viability and stress tolerance of probiotics—a review. Frontiers in Microbiology, 12, 818468. 35154042 10.3389/fmicb.2021.818468 PMC8829321

[ref151] Worametrachanon S. Apichartsrangkoon A. Chaikham P. Van den Abbeele P. Van de Wiele T. Wirjantoro T. I. (2014). Effect of encapsulated *Lactobacillus casei* 01 along with pressurized-purple-rice drinks on colonizing the colon in the digestive model. Appl. Microbiol. Biotechnol. 98, 5241–5250. doi: 10.1007/s00253-014-5624-8, 24615387

[ref152] Wu T. Chu X. Cheng Y. Tang S. Zogona D. Pan S. . (2021). Modulation of gut microbiota by *lactobacillus casei* fermented raspberry juice *in vitro* and *in vivo*. Foods 10:3055. doi: 10.3390/foods10123055, 34945605 PMC8702086

[ref153] Wu T. Guo S. Kwok L. Y. Zhang H. Wang J. (2025). Temperature-dependent metabolic interactions between *Streptococcus thermophilus* and *Lactobacillus delbrueckii* ssp. *bulgaricus* in milk fermentation: insights from gas chromatography–ion mobility spectrometry metabolomics. J. Dairy Sci. 108, 242–256. doi: 10.3168/jds.2024-25153, 39343235

[ref154] Yahsi Y. Sengun I. (2024). Development of fruit-based drinks fortified with probiotics, *Spirulina platensis* and pea protein. J. Food Meas. Charact. 18, 1–17. doi: 10.1007/s11694-024-02879-y

[ref155] Yan M. Yuan B. Xie Y. Cheng S. Huang H. Zhang W. . (2020). Improvement of postharvest quality, enzymes activity and polyphenoloxidase structure of postharvest *Agaricus bisporus* in response to high voltage electric field. Postharvest Biol. Technol. 166:111230. doi: 10.1016/j.postharvbio.2020.111230

[ref156] Yang D. Zhang Y. Zhao L. Wang Y. Rao L. Liao X. (2021). Pressure-resistant acclimation of lactic acid bacteria from a natural fermentation product using high pressure. Innov. Food Sci. Emerg. Technol. 69:102660. doi: 10.1016/j.ifset.2021.102660

[ref9010] Yao M. Xie J. Du H. McClements D. J. Xiao H. Li L. (2020). Progress in microencapsulation of probiotics: A review. Comprehensive Reviews in Food Science and Food Safety, 19, 857–874. doi: 10.1111/1541-4337.12532 33325164

[ref157] Yuan Y. Wang R. Wu X. Mu D. Guo L. Li X. (2025). Improving the flavor, bioactivity, and metabolic characteristics of corn silk by probiotic co-fermentation. Food Biosci. 68:106681. doi: 10.1016/j.fbio.2025.106681

[ref158] Zahid H. F. Ali A. Legione A. R. Ranadheera C. S. Fang Z. Dunshea F. R. . (2023). Probiotic yoghurt enriched with mango peel powder: biotransformation of phenolics and modulation of metabolomic outputs after *in vitro* digestion and colonic fermentation. Int. J. Mol. Sci. 24:8560. doi: 10.3390/ijms24108560, 37239906 PMC10218215

[ref159] Zahidah I. Bölek S. Terzioğlu Ö. T. Adıgüzel S. (2024). Determination of the effects of novel paraprobiotic supplement of *Lactobacillus plantarum* on soy dairy-free beverage by physicochemical, antioxidant, sensory analyses, and Raman spectroscopy technique. J. Food Sci. 89, 7189–7202. doi: 10.1111/1750-3841.17365, 39349981

[ref160] Zhang L. Taal M. A. Boom R. M. Chen X. D. Schutyser M. A. (2018). Effect of baking conditions and storage on the viability of *Lactobacillus plantarum* supplemented to bread. LWT 87, 318–325. doi: 10.1016/j.lwt.2017.09.005

[ref161] Zhang Y. Wang R. Wen Q. H. Rahaman A. Zeng X. A. (2022). Effects of pulsed electric field pretreatment on mass transfer and quality of beef during marination process. Innov. Food Sci. Emerg. Technol. 80:103061. doi: 10.1016/j.ifset.2022.103061

[ref162] Zhang Z. Yin B. Liu F. Zhou W. Wang M. Chang Z. . (2024). Effect of the initial pH of the culture medium on the nutrient consumption pattern of *Bifidobacterium animalis* subsp. *lactis* Bb12 and the improvement of acid resistance by purine and pyrimidine compounds. J. Appl. Microbiol. 135:lxae022. doi: 10.1093/jambio/lxae022 38299790

[ref163] Zhang X. Zheng Y. Liu Z. Su M. Wu Z. Xu X. (2025). Integrated analysis of characteristic volatile flavor formation mechanisms in probiotic co-fermented cheese by untargeted metabolomics and sensory predictive modeling. Food Res. Int. 211:116379. doi: 10.1016/j.foodres.2025.116379, 40356103

[ref164] Zong L. Qu H. Wang W. Chen D. Wa Y. Huang Y. . (2025). Effect of key flavor compounds in fermented soymilk on sensory attributes: integrating electronic sensory technology with GC-MS analysis. Food Chemistry: X 29:102750. doi: 10.1016/j.fochx.2025.102750, 40686865 PMC12275961

